# Identification and validation of a seven-gene metastasis-associated prognostic model in breast cancer

**DOI:** 10.3389/fgene.2026.1770418

**Published:** 2026-05-11

**Authors:** Yuan Yao, Yunsheng Zheng, Jiancong Xie, Honghao Liu, Chen Chen, Jie Cao, Ting-Ting Yin

**Affiliations:** Department of General Surgery, Guangzhou First People’s Hospital, Institute of Digestive Diseases, South China University of Technology, Guangzhou, Guangdong, China

**Keywords:** biomarkers, breast cancer, metastases, prognostic signature, tumor microenvironment

## Abstract

**Introduction:**

Metastasis is the primary cause of mortality in patients with breast cancer. This study aimed to develop a prognostic signature based on metastasis- and cancer-associated differentially expressed genes (M-CA-DEGs) and to identify potential novel therapeutic genes.

**Materials and methods:**

Data were acquired from the TCGA-BRCA, AURORA US Network, SCAN-B, and GEO databases. M-CA-DEGs were identified, and a prognostic risk model was constructed via univariate Cox and LASSO regression analyses. The prognostic value was verified using calibration curves and decision curve analysis (DCA). Independent prognostic factors were subsequently validated. Functional enrichment was assessed through GSEA and ssGSEA. Immune infiltration and mutation profiles were compared between risk groups. A TF–mRNA regulatory network was constructed. Single-cell analysis was performed to characterize gene expression patterns. The mRNA levels of prognostic genes were examined in MCF-10A mammary epithelial cells and breast cancer cell lines.

**Results:**

We developed and validated a novel seven-gene, metastasis-associated prognostic signature (*IGJ*, *CXCL14*, *PTGER3*, *RTN1*, *EGOT*, *TLR10*, *PANX2*) for breast cancer. The risk score emerged as a powerful independent prognostic factor. The low-risk group exhibited superior survival, an immunologically “hot” phenotype with enriched activated CD8^+^ T cells, and higher immune activity, whereas the high-risk group showed T-cell exclusion and enrichment in kinase signaling and metabolism. Somatic mutation landscapes differed significantly between groups. Crucially, we identified two previously under-characterized genes (*RTN1* and *TLR10*) as potential novel drivers of tumor progression. Single-cell transcriptomics unveiled their cell type-specific expression patterns, and *in vitro* assays confirmed differential expression in cancer cell lines.

**Conclusion:**

This study establishes a robust, biologically grounded, metastasis-related seven-gene prognostic model for breast cancer. Beyond prediction, our work identifies two novel therapeutic targets and reveals distinct immune and metabolic phenotypes across risk groups, thereby providing novel mechanistic insights into tumor heterogeneity and actionable targets for future therapies.

## 1 Background

Breast cancer remains a major global health challenge, ranking as the most commonly diagnosed cancer and a leading cause of cancer-related mortality among women worldwide (Bray et al., 2024). Despite advances in early detection and treatment, metastasis continues to account for the vast majority of breast cancer-associated deaths. Approximately 20%–30% of patients initially diagnosed with early-stage disease eventually develop metastatic recurrence (Shao and Varamini, 2022). The most common sites of breast cancer metastasis include bone, liver, lungs, and brain (Gerratana et al., 2015).

The clinical and molecular heterogeneity of breast cancer further complicates prognosis and therapeutic decision-making. Conventionally classified into PAM50 subtypes—Basal-like, HER2-enriched, Luminal A, and Luminal B—these categories exhibit distinct metastatic risks and outcomes. In particular, the Basal-like and HER2-enriched subtypes are associated with increased metastatic potential and poorer outcomes, whereas the Luminal A subtype is associated with more favorable survival outcomes, underscoring the need for more precise prognostic tools (Park et al., 2022).

Molecular profiling has enabled the development of gene signatures that predict clinical behavior and metastasis. However, many existing models lack validation across multiple cohorts or do not adequately incorporate the immune and mutational context of tumors. Integrating multiomics approaches—including bulk, single-cell, and spatial transcriptomics—offers new opportunities to identify robust biomarkers with biological and clinical relevance.

In this study, we developed and validated a novel metastasis-associated gene signature aimed at improving risk stratification in patients with breast cancer. Using RNA sequencing data from TCGA-BRCA and the AURORA US Network, we identified metastasis- and cancer-associated differentially expressed genes (M-CA-DEGs). Then, we applied Cox and least absolute shrinkage and selection operator (LASSO) regression to construct a seven-gene prognostic model. We validated the independent prognostic predictive ability of the risk score derived from this prognostic model. We further examined the immune microenvironment, tumor immune dysfunction and exclusion (TIDE) scores, mutation profiles, and pathway activities across risk groups. The expression patterns of the prognostic genes were validated via single-cell transcriptomics, along with *in vitro* assays in MCF-10A mammary epithelial cells and breast cancer cell lines. The seven prognostic genes are highly expressed in tumor cells, stromal cells, and immune cells, respectively. Two novel genes (*RTN1* and *TLR10*) were identified as negative regulators of breast cancer pathogenesis. Our results provide a comprehensive and clinically applicable prognostic tool that reflects the biological complexity of breast cancer metastasis.

## 2 Methods

### 2.1 Identification of M-CA-DEGs

RNA-seq data and clinical information for patients with breast invasive carcinoma (BRCA) were obtained from the TCGA-BRCA cohort via the UCSC Xena database. The dataset included 1,097 tumor samples (76 metastatic and 1,021 nonmetastatic) and 114 normal samples. Metastatic samples included those with detected metastasis at initial diagnosis and those with distant metastasis developed during follow-up. Among the tumor samples, 1,081 contained complete survival information. Additionally, RNA-seq data from the AURORA US Network (Garcia-Recio et al., 2023) were downloaded from the Gene Expression Omnibus (GEO) under accession number GSE209998, which comprised 79 metastatic and 44 primary tumor samples.

Differential expression analysis between metastatic and nonmetastatic samples in the TCGA-BRCA cohort was conducted via the “limma” package, which yielded TCGA metastasis-related DEGs (TCGA-M-DEGs). Genes with |log_2_FC| > 0.5 and nominal P < 0.05 were considered significantly differentially expressed. Similarly, DEGs between primary tumors and paratumor tissues were identified via the same method and denoted AURORA metastasis-related DEGs (AURORA-M-DEGs). Genes with |log_2_FC| > 0.5 and adjusted P < 0.05 were considered significantly differentially expressed. The intersection between TCGA-M-DEGs and AURORA-M-DEGs was subsequently identified via the jVenn tool (Bardou et al., 2014) to define a set of M-DEGs. DEGs between tumor and paratumor tissues in the TCGA-BRCA cohort were identified and denoted cancer-associated DEGs (CA-DEGs). Finally, the intersection between M-DEGs and CA-DEGs was identified via the jVenn tool to define a set of M-CA-DEGs.

### 2.2 Establishment of the prognostic model based on M-CA-DEGs

RNA-seq data and clinical information from the Sweden Cancerome Analysis Network - Breast (SCAN-B) cohort (Brueffer et al., 2018) were obtained from the GEO dataset GSE96058, with corresponding survival information retrieved from GSE81540. The GSE96058 dataset includes 3,273 tumor samples and 136 replicates. The RNA-seq data of GSE96058 contain two platforms: GSE96058-GPL11154 and GSE96058-GPL18573. GSE96058-GPL11154 was used as the training cohort (n = 3,069), while GSE96058-GPL18573 served as the validation cohort (n = 340).

The prognostic value of the M-CA-DEGs was first assessed via univariate Cox regression analysis in the SCAN-B training cohort. Genes with consistent prognostic significance (q value <0.05) were transformed into z-scores and further subjected to feature selection using the LASSO regression method (Simon et al., 2011) within the SCAN-B training cohort, using 10-fold cross-validation to select the optimal penalty parameter (λ). The λ value of 0.0059 was chosen to identify the final set of prognostic genes. A prognostic risk score was subsequently computed for each patient on the basis of a linear combination of the expression levels of selected genes weighted by their regression coefficients. Using the median risk score as the cutoff, patients were stratified into low- and high-risk groups. The predictive performance of the gene signature was evaluated via Kaplan–Meier survival analysis and time-dependent receiver operating characteristic (ROC) curves. The concordance index (C-index) was calculated to quantitatively assess the predictive performance and discriminative ability of the prognostic model. To evaluate the predictive accuracy and clinical net benefit of the constructed prognostic model, calibration curves and decision curve analysis (DCA) were performed for validation.

To enhance the robustness and stability of our prognostic signature, we benchmarked the standard LASSO-Cox framework against two alternative strategies: Adaptive LASSO and PCA-weighted LASSO. For Adaptive LASSO, weights derived from Ridge regression were applied to penalize individual coefficients, effectively capturing potential hierarchical effects. For PCA-weighted LASSO, principal component analysis was performed on the expression matrix (retaining ≥95% cumulative variance) prior to LASSO modeling to account for collinearity. Model performance was evaluated using 10-fold nested cross-validation to avoid overfitting.

The risk model was externally validated in the TCGA-BRCA and GSE96058-validation cohorts.

### 2.3 Independent prognostic analysis and nomogram construction

Univariate and multivariate Cox regression analyses were performed via GraphPad Prism 10 to evaluate the prognostic value of the risk score along with other clinical variables. Variables identified as independent prognostic factors were incorporated into a nomogram to predict 1-, 3-, and 5-year survival probabilities in BRCA patients. Calibration curves and time-dependent ROC curves were generated to assess the accuracy and reliability of the nomogram predictions. We performed validation in both the GSE96058 training cohort and the TCGA-BRCA cohort.

### 2.4 Validation of the prognostic model in distinct molecular and treatment subgroups

According to the PAM50 classification and treatment information from the GSE96058 training cohort and the TCGA-BRCA cohort, patients were divided into different subgroups (with n > 50 in each group). Kaplan-Meier survival curves, C-index calculation, and decision curve analysis (DCA) were performed to validate the performance of the prognostic model.

### 2.5 Elastic-net Cox regression-derived prognostic models

We constructed a baseline risk score using an elastic-net Cox regression model with optimal parameters (α = 0.4, λ = 0.00793) determined by 10-fold cross-validation and grid search. After quality control, 3,037 samples from the GSE96058 dataset were included as the training cohort, and 10 clinical features with non-zero coefficients were screened to build the risk score. We further evaluated four prognostic models (Model A: clinical variables; Model B: clinical + PAM50; Model C: clinical + 7-gene signature; Model D: elastic-net-based clinical risk score) in the GSE96058 validation cohort. Model performance was assessed by C-index, time-dependent AUC, calibration curves, and decision curve analysis (DCA) at 3- and 5-year time points.

### 2.6 Functional enrichment analysis in risk-stratified groups

Gene set enrichment analysis (GSEA, version 4.4.0) was employed to perform Kyoto Encyclopedia of Genes and Genomes (KEGG) and Gene Ontology (GO) enrichment analyses to compare low- and high-risk groups within the TCGA-BRCA cohort. Gene sets with FDR < 0.05 were considered to be significantly enriched.

Additionally, the z score-normalized ssGSEA scores for 1,383 constituent pathways across 1,066 TCGA-BRCA samples were obtained from the UCSC Xena database.

### 2.7 Immune microenvironment analysis

To investigate immune heterogeneity between the low- and high-risk groups in the TCGA-BRCA cohort, we quantified the enrichment levels of 28 immune cell types (Bindea et al., 2013) via the single-sample gene set enrichment analysis (ssGSEA). Immune infiltration estimates for the TCGA-BRCA cohort were obtained from TIMER 2.0 (Li et al., 2020). Additionally, to evaluate potential differences in immunotherapy response between risk groups, we utilized the TIDE framework (Jiang et al., 2018) to estimate scores for TIDE, dysfunction, and exclusion, as well as MSI expression signature, IFN-γ, Merck18, CD8, MDSC, CAF, and TAM M2.

Data on tumor mutation burden, clonal, clonal-neo, sub-clonal, subclonal-neo, ploidy, and purity for TCGA-BRCA were downloaded from The Cancer Immunome Atlas (TCIA) (Charoentong et al., 2017).

The GSE173839 dataset comprises 71 patients with high-risk, HER2-negative, stage II/III breast cancer who were treated with durvalumab (a PD-L1 inhibitor) and olaparib (a poly ADP-ribose polymerase inhibitor) (Pusztai et al., 2021). We downloaded the gene expression microarray data (normalized at the gene level) and relevant signature data. We then compared the differences in immune cell subsets and therapeutic predictive signatures between the low- and high-risk groups.

### 2.8 Analysis of somatic mutations across risk groups

Somatic mutation data for the TCGA-BRCA cohort, comprising 791 samples with 40,544 identifiers, were obtained from the UCSC Xena database. Differences in mutation frequencies between risk groups were assessed via the chi-square test. The Benjamini–Hochberg method was applied to adjust for multiple testing, and a q-value <0.05 was considered indicative of a significant difference in mutation frequency.

### 2.9 Prediction of transcription factors that target prognostic genes

Transcription factors (TFs) associated with the prognostic genes were predicted via the TRRUST database (Transcriptional Regulatory Relationships Unraveled by Sentence-based Text mining) (Han et al., 2018). Correlations between prognostic genes and their predicted regulatory TFs were assessed via linear regression analysis.

### 2.10 Single-cell transcriptomic profiling of prognostic genes

Single-cell RNA-seq data of the prognostic genes in breast cancer were retrieved from the Spatial Transcript Omics DataBase (STOmicsDB) (Xu et al., 2024). The single-cell dataset (accession: STDS0000346; original GEO accession: GSE225600 (Liu et al., 2023)), comprising 81,683 cells derived from primary tumors and paired metastatic lymph nodes of 4 breast cancer patients, was used to characterize gene expression patterns at cellular resolution.

To verify the expression patterns of the prognostic genes, we performed validation using the Human Breast Cancer Single Cell Atlas project (Chen et al., 2026) in CZ CELLxGENE Discover (Program et al., 2025). This atlas comprises a total of 621,200 cells across 138 patients from various breast cancer subtypes, including 242,613 cells in the Epithelial Compartment, 274,555 cells in the Immune Compartment, and 104,032 cells in the Stromal Compartment.

Based on the single-cell RNA-sequencing results of seven prognostic genes, we divided them into three gene sets: immune, malignant tumor, and stromal. We then evaluated the prognostic performance of prognostic models constructed using individual gene sets and their combinations. The evaluation was performed using the C-index and time-dependent AUC values at 1, 3, and 5 years.

### 2.11 Cell culture

The human mammary epithelial cell line MCF-10A (RRID: CVCL_0598) and breast cancer lines MCF-7 (RRID: CVCL_0031), SKBR3 (RRID: CVCL_0033), MDA-MB-231 (RRID: CVCL_0062) and MDA-MB-468 (RRID: CVCL_0419) were purchased from Procell. MCF-10A cells were maintained in MCF-10A cell complete medium (Procell, CM-0525) (DMEM/F12 + 5% house serum + 20 ng/mL EGF + 0.5 μg/mL hydrocortisone +10 μg/mL insulin + 1% NEAA + 1% P/S). MCF-7 cells were cultured in DMEM (Thermo Scientific, C11995500BT) supplemented with 10% fetal bovine serum (FBS; ExCell Bio, FSP500) plus 1% penicillin‒streptomycin antibiotic solution (Beyotime, C0222) with 10 μg/mL insulin (Asegene, PB180432). SKBR3 cells were cultured in DMEM supplemented with 10% FBS plus 1% P/S. MDA-MB-231 and MDA-MB-468 cells were maintained in Leibovitz’s L-15 medium (iCell, iCell-0009) supplemented with 10% FBS plus 1% P/S at 37 °C in air. MCF-10A, MCF-7 and SKBR3 cells were incubated at 37 °C in 5% CO_2_.

### 2.12 Gene expression of different isoforms

Isoform-level expression data for genes in the TCGA-BRCA cohort were obtained from the TCGA Splicing Variants Database (TSVdb) (Sun et al., 2018).

### 2.13 cDNA synthesis and qPCR analysis

RNA was extracted via an EZ-press RNA Purification Kit (EZBioscience, B0004D). The genomic DNA was removed, and 1 μg of RNA was converted to cDNA via the Color All-in-one Reverse Transcription Kit (with DNase) (EZBioscience, RT3C). Real-time reverse transcription PCR was performed via the use of 2×SYBR Green Color qPCR Mix (ROX2 plus) (EZBioscience, A0012-R2) on a QuantStudio™ 5 Real-Time PCR System (Roche). The expression levels of the target genes were normalized relative to those of 18S rRNA via the ΔΔCt method. Electrophoretograms of qPCR products were generated to confirm gene expression (Additional File 1). The qPCR primers used are listed in Supplementary Table S1.

### 2.14 Statistical analysis

The data from different groups were compared via the Student’s unpaired t-test, the chi-square test, or one-way ANOVA. P values in Kaplan‒Meier curves were calculated via the log-rank test method. A P or Q value less than 0.05 was considered statistically significant. Graphs were created via GraphPad Prism 10, Sangerbox webtool (Shen et al., 2022) or R.

## 3 Results

### 3.1 Screening and validation of a novel gene signature (M-CA-DEGs) for breast cancer metastasis

**FIGURE 1:**
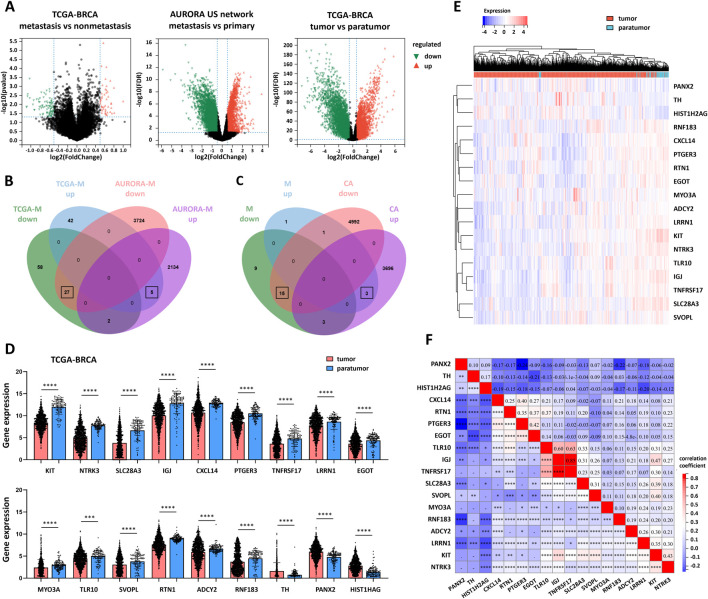
Screening of metastasis-related genes in BRCA. **(A)** Differentially expressed genes (DEGs) between the metastasis and nonmetastasis groups in the TCGA-BRCA cohort and the metastasis and primary groups in the AURORA US Network, tumor and para-tumor groups in the TCGA-BRCA cohort. **(B)** M-DEGs identified using DEGs of the metastasis and nonmetastasis groups in the TCGA-BRCA, metastasis and primary groups in the AURORA US Network. **(C)** M-CA-DEGs identified via M-DEGs and DEGs between tumors and paratumor tissues in the TCGA-BRCA cohort. **(D)** Expression of M-CA-DEGs between tumors and paratumor tissues in the TCGA-BRCA cohort. **(E)** Heatmap and clustering analysis of M-CA-DEGs between tumors and paratumor tissues in TCGA-BRCA cohort via Euclidean distance and the complete linkage method. **(F)** Correlation heatmap of M-CA-DEGs in tumor samples from TCGA-BRCA. The data are presented as the means ± SDs. P values were calculated via Student’s unpaired t-test in **(D)** and Spearman correlation analysis in **(F)**. *P < 0.05; **P < 0.01; ***P < 0.001; ****P < 0.0001.

To identify genes involved in breast cancer metastasis, we defined metastasis- and cancer-associated differentially expressed genes (M-CA-DEGs). In the TCGA-BRCA cohort, we identified 134 metastasis-related DEGs (TCGA-M-DEGs) between metastatic and nonmetastatic samples, of which 47 were upregulated and 87 were downregulated (Figure 1A). Similarly, analysis of the AURORA US Network dataset revealed 5,892 metastasis-related DEGs (AURORA-M-DEGs) between metastatic and primary breast tumors, including 2,141 upregulated and 3,751 downregulated genes (Figure 1A). By intersecting the TCGA-M-DEGs and AURORA-M-DEGs, we obtained 32 common M-DEGs (5 upward and 27 downward) (Figure 1B). Additionally, we identified 8,710 cancer-related DEGs (CA-DEGs) between breast cancer and para-tumor samples from the TCGA-BRCA cohort, comprising 3,702 upregulated and 5,008 downregulated genes (Figure 1A). Finally, the overlap between the M-DEGs and CA-DEGs yielded 18 M-CA-DEGs (15 upregulated and 3 downregulated) (Figure 1C).

We further evaluated the expression patterns of the 18 M-CA-DEGs in tumor versus para-tumor tissues within the TCGA-BRCA cohort. The expression profiles are presented as column tables (Figure 1D) and heatmaps (Figure 1E). Additionally, correlation analysis of these 18 M-CA-DEGs revealed that the majority were significantly coexpressed (Figure 1F).

The differential expression of M-CA-DEGs was further validated in both the TCGA-BRCA cohort (tumor vs. paratumor; Supplementary Figures S1A,C) and the AURORA US Network dataset (metastatic vs. primary tumors; Supplementary Figures S1B,D), with results consistent with the differential expression analysis.

### 3.2 Construction and validation of a prognostic risk model based on M-CA-DEGs

**FIGURE 2:**
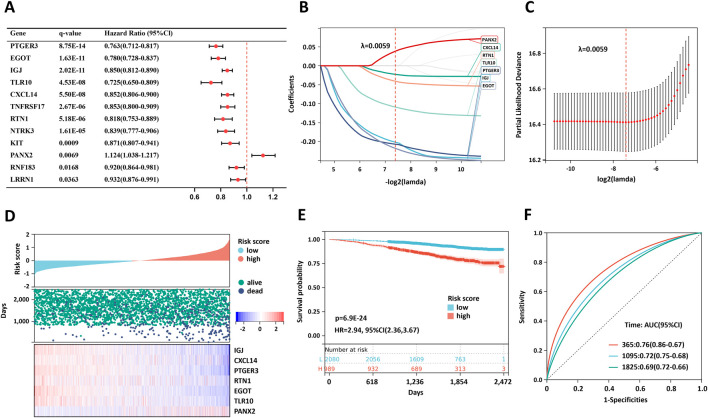
Construction of the prognostic model in BRCA. **(A)** Univariate Cox analysis of twelve prognostic genes in the SCAN-B training set. **(B,C)** LASSO-Cox analysis. **(D)** Heatmap of the risk score and prognostic genes. **(E)** Kaplan‒Meier curve of the prognostic model. **(F)** ROC curve of the prognostic model. P values were calculated via the log-rank test.

To evaluate the prognostic significance of the 18 M-CA-DEGs, we performed univariate Cox regression analysis in the SCAN-B training set and found 12 genes (*PTGER3*, *EGOT*, *IGJ*, TLR10, *CXCL14*, *TNFRSF17*, *RTN1*, *NTRK3*, *KIT*, *PANX2*, *RNF183*, and *LRRN1*) were significantly associated with prognosis (q < 0.05) (Figure 2A).

Subsequently, LASSO regression analysis was applied to refine the gene set, leading to the selection of seven prognostic genes (*IGJ*, *CXCL14*, *PTGER3*, *RTN1*, *EGOT*, *TLR10*, *PANX2*) for constructing a risk prediction model in the SCAN-B cohort (Figures 2B,C).

The risk score was calculated via the following formula:

$$Risk\;score=IGJ\times \left(-0.2018\right)+CXCL14\times \left(-0.0227\right)+PTGER3\times \left(-0.2070\right)+RTN1\times \left(-0.0410\right)+EGOT\times \left(-0.2183\right)+TLR10\times \left(-0.1092\right)+PANX2\times \left(0.0388\right)$$

Benchmarking results demonstrated that both Adaptive LASSO and PCA-weighted LASSO models achieved stable convergence (Supplementary Figures S2A,B). Notably, the corrected C-index from 10-fold nested cross-validation was 0.6724 for Adaptive LASSO and 0.6740 for PCA-weighted LASSO, closely aligning with the predictive performance of our original model. These findings confirm the stability and reliability of the identified prognostic features across different mathematical frameworks.

On the basis of the median risk score, patients were classified into low-risk and high-risk groups (Figure 2D). Expression analysis revealed that six protective genes were downregulated in the high-risk group, whereas *PANX2* was upregulated (Figure 2D). Patients in the high-risk group experienced significantly worse overall survival than those in the low-risk group did (Figure 2E). The predictive performance of the gene signature was assessed via time-dependent ROC analysis, yielding AUC values of 0.76 at 1 year, 0.72 at 3 years, and 0.69 at 5 years, indicating reasonable prognostic accuracy (Figure 2F). The C-index of the prognostic model was 0.6831, reflecting its robust predictive ability.

Furthermore, the risk model was validated in the SCAN-B validation set (Supplementary Figures S3A–C) and an external cohort, TCGA-BRCA (Supplementary Figures S3D–F), with corresponding C-index values of 0.7496 and 0.6640, respectively. These findings confirm its robustness as a prognostic predictor for breast cancer (BRCA) patients.

Ultimately, we validated the calibration and clinical applicability of the prognostic model in the GSE96058 training set, GSE96058 validation set, and TCGA-BRCA cohort (Supplementary Figures S4A–C). In the GSE96058 training set, the calibration slope was 1.158, which was close to the ideal value of 1. The observed risk (0.0574) was highly consistent with the expected risk (0.0575), accompanied by an O/E ratio of 0.997 and a calibration-in-the-large value of merely −0.0026, suggesting almost no deviation between the model-predicted risk and the actual clinical risk (Supplementary Table S2). For the GSE96058 validation set, the calibration slope was 1.509, the O/E ratio was 1.001, and the calibration-in-the-large value was 0.0009, which further verified the calibration stability of the model in the homologous cohort (Supplementary Table S2). In the external TCGA-BRCA cohort, the calibration slope was 0.724, the O/E ratio was 0.968, and the calibration-in-the-large value was −0.0325. Despite a slight deviation from the ideal range, the observed risk still maintained a high consistency with the expected risk, indicating that the model retained acceptable calibration capacity in the heterologous cohort (Supplementary Table S2).

In the calibration plots of all cohorts, the scatter points were distributed closely to the ideal diagonal line, and the LOESS smoothing curve showed no obvious offset. Combined with the results of decision curve analysis (DCA) (Supplementary Figures S4D–F), these findings confirm that the model possesses favorable calibration performance and clinical decision-making value in 3-year survival risk prediction, and can provide a reliable basis for risk stratification in diverse populations.

### 3.3 The risk score serves as an independent and clinically actionable prognostic factor across molecular subtypes and therapy

Clinical characteristics (age, tumor size, lymph node status, ER status, PGR status, HER2 status, Ki67 status, Nottingham histologic grade (NHG), and PAM50 subtype) across low- and high-risk groups in the SCAN-B training set are summarized in Supplementary Table S3. Compared with patients in the low-risk group, those in the high-risk group presented distinct clinical and molecular characteristics, including advanced age, larger tumor size, lower rates of ER and PGR positivity, higher HER2 positivity, elevated Ki67 expression, and more advanced NHG stage (Supplementary Table S3). In terms of PAM50 subtyping, the high-risk group demonstrated a greater prevalence of Basal-like, HER2-enriched, and Luminal B subtypes, along with a lower proportion of Luminal A tumors (Supplementary Table S3).

In the TCGA-BRCA cohort, patients in the high-risk group were older, had larger tumors (T), had lower ER or PGR positivity rates, had higher HER2 positivity rates, and more advanced disease stages (Supplementary Table S4). The distribution of the PAM50 molecular subtypes in the high-risk group was consistent between the TCGA-BRCA cohort and the SCAN-B training set (Supplementary Tables S3, S4). Consistent with its prognostic value, the risk score distribution varied significantly among molecular subtypes. In both the SCAN-B training set and TCGA-BRCA cohort, the LumA subtype presented the lowest risk scores, whereas Basal-like, HER2-enriched, and LumB subtypes presented higher scores (Supplementary Figures S5A,B).

**FIGURE 3:**
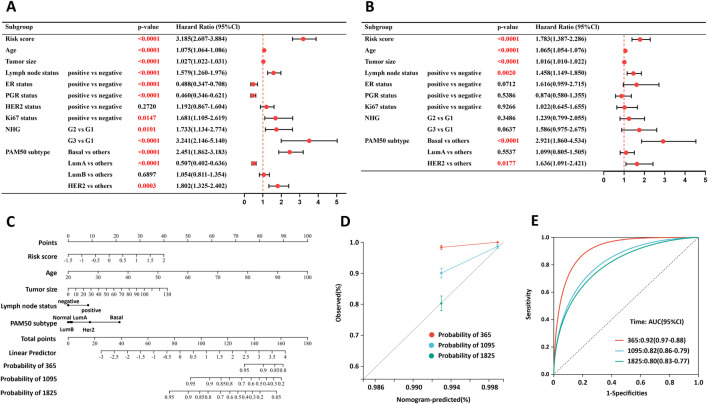
Validation of the risk score as an independent prognostic factor. Univariate **(A)** and multivariate **(B)** Cox regression analyses of prognostic factors in the SCAN-B training set. Independent prognostic factors were identified via the nomogram **(C)** and calibration curves **(D)**. **(E)** ROC curve of the nomogram. P values were calculated via the log-rank test.

Univariate Cox regression analysis indicated that the risk score, age, tumor size, lymph node status, ER status, PGR status, Ki67 status, NHG, and PAM50 subtype were significantly associated with prognosis (Figure 3A). Subsequent multivariate analysis confirmed that the risk score, age, tumor size, and lymph node status remained independent prognostic factors for BRCA (Figure 3B). A nomogram incorporating these independent predictors was developed to estimate survival probability (Figure 3C). The C-index of the nomogram was 0.7894 (95% CI: 0.7635–0.8153). The calibration curves demonstrated satisfactory agreement between the predicted and observed outcomes for 1-, 3-, and 5-year survival (Figure 3D). Time-dependent ROC analysis further supported the discriminative ability of the nomogram, with AUC values indicating robust predictive performance across these time points (Figure 3E).

We verified the independent predictive ability of the model in TCGA-BRCA, and the C-index of the nomogram was 0.7486 (Supplementary Figure S6).

We further assessed the prognostic utility of the risk score across PAM50 molecular subtypes (Supplementary Figures S7A,C). In the SCAN-B cohort, lower risk scores predicted favorable survival across all subtypes (Supplementary Figure S7A), with corresponding C-index values of 0.6335 for basal-like tumors, 0.6445 for HER2-enriched tumors, 0.6680 for LumA tumors, and 0.5222 for LumB tumors. In the TCGA-BRCA cohort, lower risk scores were consistently correlated with improved survival in HER2-enriched, LumA, and LumB subtypes, while showing a non-significant trend in the basal-like subtype (Supplementary Figure S7C). The respective C-index values were 0.5616 for basal-like, 0.6978 for HER2-enriched, 0.6256 for Luminal A, and 0.6938 for Luminal B subtypes.

Next, we evaluated the prognostic predictive value of the risk score across distinct treatment subgroups (Supplementary Figures S7B,D). In the SCAN-B cohort, a low risk score was consistently associated with favorable survival in both endocrine therapy-treated and -untreated patients, as well as in chemotherapy-treated and -untreated subgroups (Supplementary Figure S7B). The corresponding C-index values were 0.6660 (endocrine therapy-untreated), 0.6692 (endocrine therapy-treated), 0.6996 (chemotherapy-untreated), and 0.6856 (chemotherapy-treated). In the TCGA-BRCA cohort, a low-risk score was consistently linked to favorable survival in both radiation-treated and -untreated patients, as well as in the targeted molecular therapy-treated subgroup (Supplementary Figure S7D). The corresponding C-index values were 0.6757 (radiation-treated), 0.6517 (radiation-untreated), and 0.6548 (targeted molecular therapy-untreated).

Collectively, these results indicate that the risk score retains robust prognostic value across all PAM50 subtypes and treatment subgroups.

### 3.4 Subtype utility validation of the risk score and comparative analysis of multiple prognostic models

In the TCGA-BRCA dataset, we further evaluated the clinical utility of the risk score according to PAM50 molecular subtypes (Supplementary Figure S8). Subgroup analysis revealed distinct differences in the net clinical benefit of the risk score across PAM50 subtypes. Specifically, in Luminal B, HER2-enriched, and basal-like subtypes, the risk score-guided therapy demonstrated a significantly higher net benefit than the strategies of “treating all patients” or “treating no patients”, indicating a clear clinical utility. In contrast, in Luminal A subtypes, the net benefit of the risk score largely overlapped with that of the “treating all” strategy, suggesting limited clinical gain.

Subsequently, we constructed a baseline risk score in the training set using the elastic-net Cox regression model, and developed four distinct models as follows: Model A incorporated only clinical covariates, including age, tumor size, lymph node status, histological grade, and ER/PR/HER2/Ki67 status; Model B integrated clinical variables with the PAM50 classification; Model C combined clinical covariates with the 7-gene signature; and Model D was based on the linear predictive score of clinical variables screened by the elastic-net Cox model, calculated using pre-trained model parameters.

We then validated the prognostic predictive performance of the four models—Model A (clinical variables only), Model B (clinical + PAM50), Model C (clinical + 7-gene signature), and Model D (clinical + elastic-net model)—in the GSE96058 validation set. The C-index, AUC, calibration curve analysis, and decision curve analysis (DCA) were performed to evaluate model performance.

In terms of discriminatory ability, Model C (clinical + 7-gene signature) and Model B (clinical + PAM50) exhibited the optimal performance, with C-index values of 0.867 and 0.865, respectively (Supplementary Table S5). Model A, which contained only clinical covariates, also showed robust discriminatory ability with a C-index of 0.858, serving as a reliable baseline model. In contrast, Model D (clinical + elastic-net model) had a markedly low C-index of 0.767, demonstrating significantly insufficient discriminatory capacity (Supplementary Table S5).

Calibration analysis revealed that the 3-year calibration curves of Model A, Model B, and Model C were all closely distributed around the ideal diagonal line, indicating excellent consistency between the predicted and actual survival risks (Supplementary Figure S9A). Conversely, Model D displayed relatively obvious calibration bias (Supplementary Figure S9A). Further decision curve analysis (DCA) demonstrated that within the 3-year risk threshold range of 0–0.20, the net benefit curves of Model A, Model B, and Model C were significantly higher than those of the “treat all” and “treat none” strategies, highlighting remarkable clinical utility (Supplementary Figure S9B). By comparison, Model D yielded an overall low net benefit, suggesting limited clinical application value (Supplementary Figure S9B).

Collectively, Model C (clinical + 7-gene signature) and Model B (clinical + PAM50) were significantly superior to the other models in terms of discriminatory ability, calibration degree, and net clinical benefit, representing more promising prognostic predictive tools for clinical practice.

### 3.5 Divergent functional enrichment profiles underlie risk stratification in breast cancer

**FIGURE 4:**
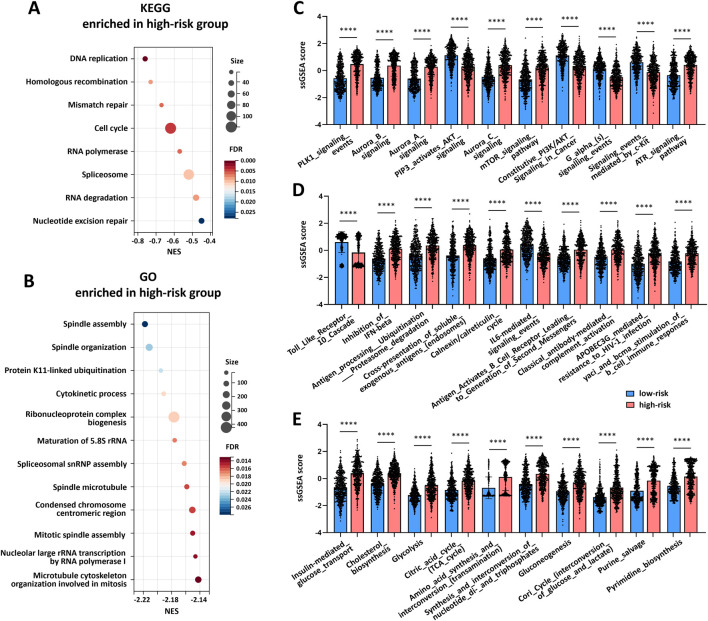
Functional enrichment in the low-risk and high-risk groups. The top 12 most significantly differentially expressed KEGG pathways **(A)** and GO **(B)** in the TCGA low-risk (n = 541) and high-risk (n = 540) groups. The ssGSEA scores of the top 10 most significantly differentially expressed molecular signaling pathways **(C)**, immune-related pathways **(D)** and metabolic pathways **(E)** are displayed for the TCGA low-risk (n = 530) and high-risk (n = 521) groups. The data are presented as the means ± SDs. Q values were calculated via GSEA. P values were calculated via Student’s unpaired t-test in **(C–E)**. ****P < 0.0001.

We performed KEGG and GO enrichment analyses to compare biological functions between the low-risk and high-risk groups within the TCGA-BRCA cohort. All significantly enriched KEGG pathways and the top 12 significantly enriched GO terms are presented in Figures 4A,B, respectively. The high-risk group was mainly enriched in pathways governing cell proliferation and mitosis, DNA replication and repair, and RNA transcription and processing.

Furthermore, we compared the ssGSEA scores of 1,387 gene sets between the risk groups. The top 10 significantly differentially expressed molecular signaling, immune-related, and metabolic pathways are presented in Figures 4C–E, respectively. The high-risk group presented increased activity of kinase-related genes such as PLK1, Aurora, mTOR, and ATR, which are involved in tumor progression (Figure 4C). The high-risk group exhibited enhanced activity in antigen presentation (Figure 4D). Additionally, the high-risk group presented increased enrichment of the TCA cycle, glycolysis, gluconeogenesis, and amino acid synthesis/transport pathways, suggesting that metabolic activation may contribute to increased tumor risk (Figure 4E).

### 3.6 Distinct immune microenvironment landscapes underlie low- and high-risk breast cancer

**FIGURE 5:**
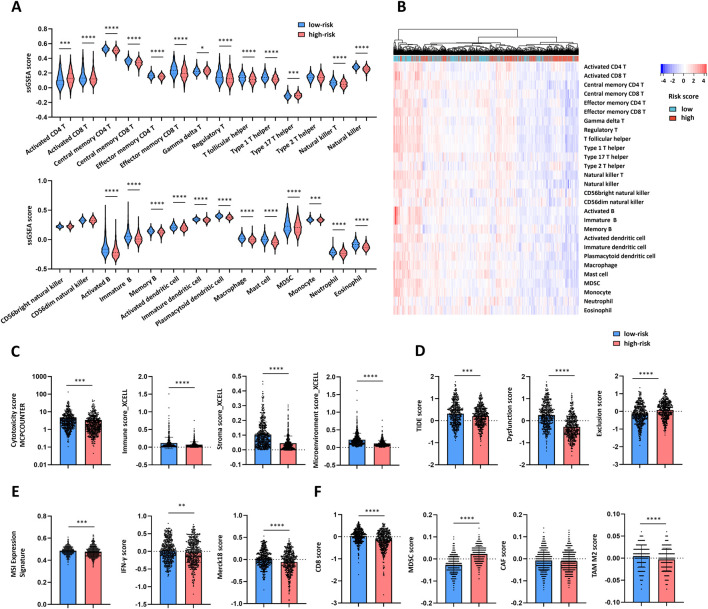
Immune landscape in the low-and high-risk groups. **(A)** Cell infiltration of 28 immune cell types between the low-risk (n = 540) and high-risk (n = 541) groups in the TCGA-BRCA database was compared via the ssGSEA method. **(B)** Heatmap of 28 immune cell types between the low-risk and high-risk groups via Euclidean distance and the complete linkage method. **(C)** Cytotoxicity score_MCPCOUNTER, immune score_XCELL, stroma score_XCELL and microenvironment score_XCELL in the low-risk (n = 540) and high-risk (n = 539) groups. **(D)** TIDE, dysfunction and exclusion scores in the low-risk (n = 515) and high-risk (n = 504) groups. **(E)** MSI Expression signature, IFN-γ, and Merck18 scores in the low-risk (n = 515) and high-risk (n = 504) groups. **(F)** CD8, MDSC, CAF and TAM scores in the low-risk (n = 515) and high-risk (n = 504) groups. The data are presented as the means ± SDs. P values were calculated via Student’s unpaired t-test. *P < 0.05; **P < 0.01; ***P < 0.001; ****P < 0.0001.

Using the ssGSEA method, we compared the infiltration levels of 28 immune cell types between the low-risk and high-risk groups. Twenty-two types of immune cells, including activated CD8^+^ T cells, Th1 cells, NK cells, activated dendritic cells, macrophages, and neutrophils, were significantly enriched in the low-risk group (Figure 5A). A heatmap was generated to visualize the associations between risk groups and the tumor immune microenvironment (Figure 5B). Compared with the high-risk group, the low-risk group presented significantly greater cytotoxicity, stromal, immune, and microenvironment scores (Figure 5C), suggesting a more active immune microenvironment in low-risk BRCA patients.

Further analysis of TIDE, dysfunction, and exclusion scores revealed elevated TIDE and dysfunction scores in the low-risk group, whereas the high-risk group presented higher exclusion scores (Figure 5D). These results suggest that T-cell dysfunction may be the primary barrier to the immunotherapy response in the low-risk group, whereas T-cell exclusion predominates in the high-risk group. The low-risk group exhibited higher MSI expression signature, IFN-gamma score, and Merck18 score, indicating an immune-activated phenotype (Figure 5E). Additionally, the low-risk group presented increased abundances of CD8^+^ T cells and M2-type tumor-associated macrophages (TAMs), whereas the high-risk group presented increased number of myeloid-derived suppressor cells (MDSCs) (Figure 5F). This pattern suggests that M2 TAMs may promote T-cell dysregulation in low-risk patients, whereas MDSCs may contribute to T-cell exclusion in high-risk patients.

We compared the mutation burden, clonality, and neoantigen levels between the low- and high-risk groups, and no significant differences were observed, suggesting that the immune disparities between the two groups were not driven by high-frequency gene mutations (Supplementary Figure S10A). We found that ploidy and tumor purity in the low-risk group were significantly lower than those in the high-risk group, indicating that ploidy and tumor purity may contribute to the regulation of the immune microenvironment in breast cancer (Supplementary Figure S10A).

Finally, we compared immune signature scores between low- and high-risk groups in HER2-negative, stage II/III breast cancer patients treated with immunotherapy (GSE173839 dataset). The results showed that the low-risk group had higher levels of T and B cells, whereas no significant differences were observed in DC and macrophage levels between the two groups (Supplementary Figure S10B). Meanwhile, the TAMsurr_TcClassII ratio, which reflects the balance between immunosuppressive tumor-associated macrophages (TAMs) and antitumor cytotoxic T-cell immunity coupled with antigen-presentation capacity, was comparable between the two groups (Supplementary Figure S10B). No differences were found in the TIS signature or STAT1 expression either (Supplementary Figure S10C). These findings suggest that although the low-risk group exhibited stronger immune infiltration, the two groups showed similar responsiveness to immunotherapy. In addition, we found that the high-risk group displayed higher PARPi7 signature, mitotic signature, and ESR1_PGR_ave, indicating greater sensitivity to PARP inhibitors, anti-mitotic chemotherapy, and endocrine therapy (Supplementary Figure S10D).

### 3.7 Risk-associated mutational profiles and their correlation with risk scores

We characterized the mutational profiles of the low- and high-risk groups using data from the TCGA-BRCA cohort. We used the chi-square test to evaluate the difference in mutation frequencies between the low-risk and high-risk groups, and then calculated FDR-adjusted q-values. Using this approach, we identified four differentially mutated genes (Supplementary Figure S11A). Specifically, the high-risk group presented increased mutation rates in *TP53* and *SPTA1*, whereas the low-risk group presented increased mutation frequencies in *CDH1* and *PIK3CA*. Consistent with these findings, patients carrying mutations in *TP53* and *SPTA1* were associated with significantly higher risk scores than their wild-type counterparts. Conversely, mutations in *CDH1* and *PIK3CA* were correlated with lower risk scores (Supplementary Figure S11B).

### 3.8 Identification and clinical relevance of a prognostic transcription factor regulatory network

A total of six TF–mRNA regulatory pairs were predicted via TRRUST database. Within these networks, *SP1* was predicted to positively regulate *CXCL14* (Ikoma et al., 2012), whereas *FOXP3* (Bell et al., 2007), *VDR*, and *RXRA* (Verma et al., 2014) were predicted to positively regulate *TLR10*.

We performed correlation analysis of TF and target gene expression in the TCGA-BRCA cohort. Consistent with these predictions, *SP1* expression was positively correlated with *CXCL14*, while *FOXP3* and *VDR* expression were correlated positively with *TLR10* (Supplementary Figure S12A). Further analysis revealed that *SP1*, and *RXRA* were downregulated in tumor tissues compared with para-tumor samples in TCGA-BRCA, whereas *FOXP3* and *VDR* were upregulated (Supplementary Figure S12B). In the AURORA cohort, *SP1*, *FOXP3*, *VDR*, and *NPAS3* were downregulated in metastatic tumors relative to primary tumors (Supplementary Figure S12C). Survival analysis via the SCAN-B database revealed that higher expression of *SP1* and *RXRA* was associated with better prognosis (Supplementary Figure S12D).

In the *VDR*/*RXRA*-*TLR10* axis, *TLR10* expression may be influenced less by TF expression levels and potentially more by bioactive vitamin D3 availability.

### 3.9 Single-cell and spatial transcriptomic mapping reveal the cell type-specific expression of prognostic genes

**FIGURE 6:**
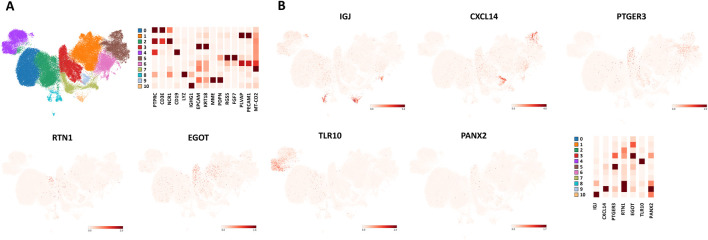
Single-cell sequencing of prognostic genes. **(A)** Clustering of BRCA single-cell sequencing data from STDS0000346 (STOmics database). **(B)** Single cell sequencing of prognostic genes.

We investigated the expression patterns of the prognostic genes via single-cell RNA sequencing and spatial transcriptomics data from the STOmicsDB. Single-cell analysis revealed major cell subsets, including T cells (Cluster 0; marker: CD3E), NK cells (Cluster 2; NCR1), B cells (Cluster 4; CD19), plasma cells (Cluster 10; IGHG1), epithelial cells (Cluster 3; EPCAM, KRT18), cancer-associated fibroblasts (CAFs; Cluster 9; MME, PDPN), perivascular-like (PVL) cells (Cluster 5; RGS5, FGF7), and tumor endothelial cells (TECs; Clusters 1 and 6; PLVAP, PECAM1) (Figure 6A).

Expression analysis revealed cell type-specific enrichment of the prognostic genes: *IGJ* was predominantly expressed in plasma cells; *CXCL14* was expressed in CAFs; *PTGER3* was expressed primarily in PVL cells, with secondary expression in epithelial cells; *RTN1* was expressed primarily in myeloid cells, with secondary expression in CAFs; *EGOT* was expressed mainly in epithelial cells and was detected in TECs; *TLR10* was expressed in B cells; and *PANX2* showed the highest expression in CAFs (Figure 6B).

We used another set of single-cell sequencing data to compare the expression of prognostic genes across different cell subsets. The expression patterns of these prognostic genes among cell subsets were consistent between the two datasets (Supplementary Table S6).

Based on these results, we classified the seven prognostic genes into three groups: immune (*IGJ*, *RTN1*, *TLR10*), stromal (*CXCL14*, *PTGER3*, *PANX2*), and malignant (*EGOT*). We then compared the prognostic performance of models constructed using each individual group and their combinations (Supplementary Table S7). The results demonstrated that the prognostic model combining all three groups achieved the best performance. The C-index and 3-year AUC of the “immune + malignant” and “immune + stromal” models were higher than those of the “malignant + stromal” model, whereas their 1-year AUC was lower. Comparison among the three single-gene groups showed that the *immune* group had the lowest 1-year AUC but the highest 3-year AUC.

These findings suggest that diverse cellular components, including tumor cells, stromal cells, and immune cells, collectively contribute to prognosis. Immune cells may have a relatively weak impact on short-term prognosis but exert a stronger influence on long-term prognosis.

### 3.10 Comprehensive expression profiling of prognostic genes across breast cancer cell lines

**FIGURE 7:**
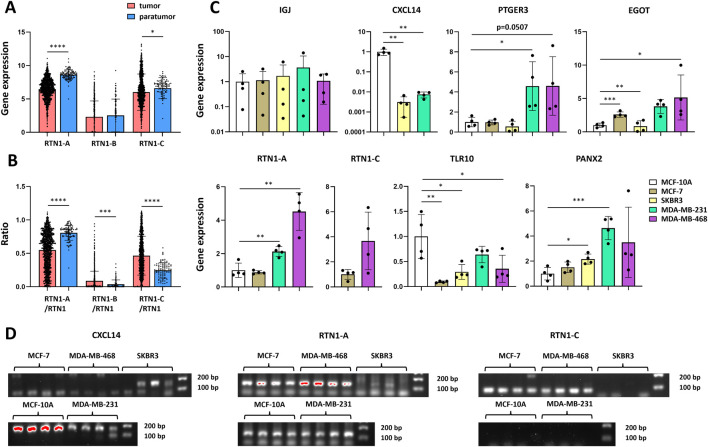
Prognostic gene expression in cell lines. **(A)** RTN1-A, RTN1-B and RTN1-C expression in tumor (n = 1,080) and para-tumor (n = 98) samples from TCGA-BRCA. **(B)** Ratios of RTN1-A/RTN1, RTN1-B/RTN1 and RTN1-C/RTN1 in tumor and paratumor samples from TCGA-BRCA. **(C)** mRNA levels of prognostic genes in MCF-10A, MCF-7, SKBR3, MDA-MB-231 and MDA-MB-468 cells (n = 4). **(D)** Electrophoretogram of the PCR products of CXCL14 (171 bp), RTN1-A (134 bp), and RTN1-C (78 bp). The data are presented as the means ± SDs. P values were calculated via Student’s unpaired t-test. *P < 0.05; **P < 0.01; ***P < 0.01; ****P < 0.0001.

We examined the expression of the eight prognostic genes in the human mammary epithelial cell line MCF-10A and the breast cancer cell lines MCF-7, SKBR3, MDA-MB-231, and MDA-MB-468. *RTN1* contains 3 isoforms produced by alternative splicing (UniProt ID: Q16799). Using the TSVdb database, we found that *RTN1-A* and *RTN1-C* were expressed in breast cancer, whereas *RTN1-B* was expressed at relatively low levels (Figure 7A). Although both isoforms were downregulated in tumor compared with paratumor tissues, *RTN1-A* was more substantially reduced (Figure 7A). Moreover, the *RTN1-A*/*RTN1* ratio was significantly lower in tumors than in normal controls, suggesting a potentially critical role for *RTN1-A* in breast cancer (Figure 7B). mRNA analysis revealed that *TLR10*, *PTGER3*, *PANX2*, *EGOT,* and *IGJ* were expressed across all the cell lines (Figure 7C). *CXCL14* was highly expressed in MCF-10A cells, expressed at low levels in SKBR3 and MDA-MB-231 cells, and was undetectable in MCF-7 and MDA-MB-468 cells (Figures 7C,D). *RTN1-A* was absent in SKBR3 cells but was expressed at significantly higher levels in MDA-MB-231 and MDA-MB-468 cells than in MCF-10A cells (Figures 7C,D). *RTN1-C* was detectable only in MCF-7 and MDA-MB-468 cells (Figures 7C,D). Additionally, *PTGER3* expression was significantly higher in MDA-MB-231 and MDA-MB-468 cells than in MCF-10A cells, whereas *PANX2* expression was significantly higher in SKBR3 and MDA-MB-231 cells than in MCF-10A cells (Figure 7C). *TLR10* was downregulated in MCF-7, SKBR3, and MDA-MB-468 relative to MCF-10A cells (Figure 7C).

## 4 Discussion

Despite significant advances in the early diagnosis and treatment of breast cancer, tumor metastasis remains the leading cause of patient mortality. This underscores the urgent need to develop reliable prognostic biomarkers that can reflect the molecular complexity of tumor progression (Park et al., 2022). In this study, we integrated multiomics data to construct and validate a seven-gene metastasis-associated prognostic signature (*IGJ*, *CXCL14*, *PTGER3*, *RTN1*, *EGOT*, *TLR10*, *PANX2*). This signature effectively stratified patients into distinct risk groups with significantly different survival outcomes and demonstrated robust predictive performance across multiple independent cohorts, all major PAM50 molecular subtypes, and various treatment groups, highlighting its broad potential for clinical application. Moreover, these genes may represent novel potential regulators of breast cancer progression, thereby expanding our understanding of metastasis-associated molecular pathways.

Among the signature genes, *IGJ*, *CXCL14*, *PTGER3*, *EGOT*, and *PANX2* play established roles in breast cancer pathogenesis. The joining chain of multimeric IgA and IgM (JCHAIN, known as IGJ) is expressed mainly on plasma cells but can also be detected in mammary epithelial cells and BRCA cell lines (Wang et al., 2023b). The overexpression of IGJ led to reduced breast cancer growth and metastasis via the suppression of NF-κB signaling (Wang et al., 2023b).

*CXCL14* overexpression suppressed proliferation, invasion, xenograft growth, and lung metastasis in MDA-MB-231 cells (Gu et al., 2012). Similarly, in a 4T1 murine model, *CXCL14* overexpression inhibited tumor progression and lung metastasis by modulating the tumor immune microenvironment, which was characterized by an increase in CD8^+^ T cells and a reduction in tumor-associated macrophages (TAMs) (Gibbs et al., 2022).

Prostaglandin E Receptor 3 (PTGER3) knockdown significantly promoted cell growth whereas its overexpression inhibited proliferation in MCF-7 and MDA-MB-231 cells (Tan et al., 2025). Recently, a study reported that chemotherapy can upregulate lipid oxidation in the PTGER3^+^ CAF subset, and these CAFs can enhance the function of CD8^+^ T cells, thereby improving the efficacy of immunotherapy (Ma et al., 2026). Our single-cell sequencing analysis revealed that PTGER3 is highly expressed in PVL and pericytes in breast cancer, and we speculate that this cell population in breast cancer may exert a similar function.

Eosinophil Granule Ontogeny Transcript (EGOT) is a long non-coding RNA (lncRNA) that has been implicated in multiple cancers (Li et al., 2021; Wang et al., 2023a). In breast cancer cell lines, *EGOT* significantly inhibits cell viability and migration by inactivating the Hedgehog pathway (Qiu et al., 2020).

As a Pannexin (Panx) channel protein that regulates cell tight junctions, PANX2 promotes cell proliferation and migration in colorectal cancer and prostate cancer (Jiang et al., 2025). *PANX2* exerts a pro-metastatic function in the highly metastatic breast cancer cell line MDA-LM2 (Fish et al., 2018).

Interestingly, although single-cell analysis showed that *IGJ*, *CXCL14*, *PTGER3*, and *PANX2* were more highly expressed in corresponding non-tumor cell subsets than in tumor cells, published studies have reported that these genes exert either tumor-suppressive (*IGJ*, *CXCL14*, *PTGER3*) or tumor-promoting (*PANX2*) functions in breast cancer cell lines. These functional effects are consistent with the results of our univariate Cox analysis.

This suggests that the prognostic genes in our model do not merely reflect the proportions of different cell subsets; rather, these genes most likely play functional roles in both tumor cells and non-tumor cells. Notably, two genes in the signature—*RTN1* and *TLR10*—have not been well characterized in breast cancer. Reticulon 1 (RTN1) is an ER-localized protein predominantly expressed in neuroendocrine cells. In addition, RTN1-A has also been detected in dendritic cells (DCs) and Langerhans cells, where its expression can be downregulated upon Toll-like receptor (TLR) stimulation in myeloid lineages (Cichon et al., 2022). Functionally, *RTN1-A* potentiates ER stress and promotes apoptosis, contributing to the progression of kidney disease (Fan et al., 2015). Similarly, in neuroectodermal tumors, the *RTN1-C* isoform enhances ER stress through the inhibition of HDAC4 (Fazi et al., 2009).

Toll-like receptor (TLR) 10 is an anti-inflammatory pattern-recognition receptor among the TLR family (Oosting et al., 2014). Polymorphisms in the *TLR10* gene have been linked to susceptibility to several cancers, including nasopharyngeal carcinoma (Zhou et al., 2006), prostate cancer (Stevens et al., 2008), and papillary thyroid carcinoma (Kim et al., 2013). Nevertheless, the precise function of TLR10 in tumorigenesis remains incompletely understood. TLR signaling not only modulates immune responses but also directly influences tumor cell behaviors—such as proliferation, survival, invasion, and stemness—largely through NF-κB activation (Ridnour et al., 2013). Consistent with these findings, TLR/NF-κB signaling has been shown to promote invasion and metastasis in breast cancer (Park et al., 2007; Yang et al., 2014). In our study, the low-risk group presented increased activity in “Toll_Like_Receptor_10_Cascade”. Moreover, *TLR10* expression was greater in the nontumorigenic mammary epithelial cell line MCF-10A than in the breast cancer lines MCF-7, SKBR3, and MDA-MB-468. These findings collectively suggest that *TLR10* may act as a tumor suppressor, potentially through the suppression of NF-κB signaling.

Notably, the proximal promoter of *TLR10* contains binding sites for both VDR and RXR-α (Verma et al., 2014). 1,25-Dihydroxyvitamin D_3_ has been shown to upregulate *TLR10* expression in human monocytic THP-1 cells (Verma et al., 2014). Given that vitamin D deficiency is associated with increased breast cancer risk (Atoum and Alzoughool, 2017), we propose that vitamin D may exert protective effects—at least in part—through transcriptional activation of *TLR10*.

The expression of these two novel genes was confirmed in breast cancer cell lines, supporting their relevance to mammary tissue biology. Notably, *TLR10* was consistently expressed across lines, whereas *RTN1* isoforms presented variable expression. Although *RTN1* and *TLR10* show higher expression in corresponding non-tumor cell subsets than in tumor cells, we hypothesize that these two genes may also exert functional roles in tumor cells, similar to *IGJ*, *CXCL14*, *PTGER3* and *PANX2*. We will examine the expression of these genes in clinical breast cancer samples and investigate the functions of the three candidate genes through *in vitro* and *in vivo* experiments following gene knockout and overexpression in cell lines.

Notably, the expression patterns and putative functions of these novel genes suggest their potential to influence breast cancer progression by modulating key signaling networks. Pathway enrichment analysis further revealed distinct underlying biological characteristics across the different risk groups. Several kinase pathways enriched in the high-risk group, including PLK1 (Montaudon et al., 2020), Aurora-A (Ingebriktsen et al., 2024), and S6K1 (Khotskaya et al., 2014), have previously been implicated in promoting breast cancer metastasis. Further analysis revealed a close association between signaling pathway alterations and mutational profiles across risk groups. The high-risk group showed marked enrichment of proliferation- and cell cycle-related pathways, which was consistent with a higher mutation burden in key driver genes such as *TP53*. In contrast, the low-risk group presented increased activity of “PIP3_activates_AKT_signaling”, which was associated with increased mutation frequencies in *PIK3CA*. The high-risk group had a higher mutation frequency of *SPTA1* (Spectrin Alpha, Erythrocytic 1). Mutations in *SPTA1* have been identified in multiple cancers including glioblastoma, colorectal cancer, and small-cell lung cancer (Gao et al., 2019). However, its function and underlying mechanism remain largely unclear. We hypothesize that mutant *SPTA1* may affect tumor metastasis and prognosis in breast cancer by modulating the cytoskeleton. Further studies are warranted to validate these hypotheses.

Our study observed that although tumors in the low-risk group exhibited higher levels of immune cell infiltration, the TIDE algorithm predicted a lower likelihood of response to immunotherapy. This apparent paradox highlights a critical complexity in the tumor immune microenvironment: the abundance of immune cells does not directly equate to the effectiveness of their anti-tumor function (Wherry and Kurachi, 2015; Jiang et al., 2018). Our analysis revealed that the primary barrier in the low-risk group stems from a higher T-cell dysfunction score, suggesting that the infiltrating effector T cells may be in an exhausted or suppressed state, unable to execute effective killing. Concurrently, the significant enrichment of M2-polarized tumor-associated macrophages (TAMs) in this group may further exacerbate local immunosuppression through the secretion of inhibitory factors. ssGSEA results showed that the antigen presentation capacity was weaker in the low-risk group, which is a manifestation of immune dysregulation. In GSE173839, the high-risk group was predicted to be more sensitive to chemotherapy and endocrine therapy, while no significant difference was predicted in immunotherapy sensitivity between the low-risk and high-risk groups. We speculate that the immune escape mechanisms differ between the two risk groups, and distinct strategies are needed to enhance their responsiveness to immunotherapy. Notably, predictions by TIDE and other tools have limitations; clinical data from immunotherapy trials are required to confirm the differences in immunotherapy responsiveness between the low-risk and high-risk groups.

This finding carries important translational implications: it indicates that for such patients, immune checkpoint inhibitors alone may have limited efficacy. Future therapeutic strategies should consider combination approaches aimed at reversing T-cell exhaustion or reprogramming macrophages to reactivate the pre-existing immune potential within the tumor microenvironment.

## 5 Conclusion

This study has achieved several key advances in the development and validation of a prognostic model for breast cancer. Firstly, regarding gene selection strategy, we innovatively defined “Metastasis- and Cancer-Associated Differentially Expressed Genes” (M-CA-DEGs). This strategy ensured that the seven-gene signature identified in our model is biologically anchored to both “carcinogenesis” and “metastasis”—two core processes—thereby granting the model stronger biological plausibility and specificity.

Secondly, in terms of validation comprehensiveness, we extended the clinical relevance of the model to single-cell transcriptomic resolution. This clearly revealed the expression patterns of these genes within specific cell types of the tumor ecosystem, such as CAFs, PVL cells, and plasma cells. Additionally, *in vitro* experiments validated their expression in mammary epithelial and breast cancer cell lines, substantially enhancing the robustness of our conclusions.

Finally, regarding the depth of biological insight, our model not only achieved risk stratification but also uncovered previously under-appreciated disease biology. We identified two genes, *TLR10* and *RTN1*, whose functions in tumors remain largely unknown. We also found that *SPTA1*, a rarely reported gene, exhibited a significantly higher mutation frequency in the high-risk group. Furthermore, the study constructed novel transcription factor regulatory networks, such as the *VDR*–*TLR10* axis, providing clues to the molecular basis of this subgroup.

In summary, this study not only delivers a rigorously validated prognostic tool but, more importantly, serves as a discovery platform that for the first time systematically highlights the existence of a potential new breast cancer subgroup dominated by neuroendocrine-immune crosstalk. This opens new perspectives for in-depth analysis of disease heterogeneity and the development of targeted therapeutic strategies.

## Data Availability

The TCGA-BRCA dataset used in this study is publicly available through UCSC xena, following links: https://xenabrowser.net/datapages/?cohort=TCGA%20Breast%20Cancer%20(BRCA)&removeHub=https%3A%2F%2Fxena.treehouse.gi.ucsc.edu%3A443. The data from the AURORA US Network are publicly available through the GEO (the following links: https://www.ncbi.nlm.nih.gov/geo/) under the accession code GSE209998. The data from SCAN-B are publicly available through the GEO under the accession codes GSE96058 and GSE81540. The data of BRCA patients with immune therapy are publicly available through the GEO under the accession codes GSE173839. The datasets generated during and/or analyzed during the current study are available from the corresponding author upon reasonable request.

## References

[B1] Atoum M. Alzoughool F. (2017). Vitamin D and breast cancer: latest evidence and future steps. Breast Cancer (Auckl) 11, 1178223417749816. 10.1177/1178223417749816 29434472 PMC5802611

[B2] Bardou P. Mariette J. Escudie F. Djemiel C. Klopp C. (2014). Jvenn: an interactive venn diagram viewer. BMC Bioinforma. 15 (1), 293. 10.1186/1471-2105-15-293 25176396 PMC4261873

[B3] Bell M. P. Svingen P. A. Rahman M. K. Xiong Y. Faubion W. A. Jr. (2007). FOXP3 regulates TLR10 expression in human T regulatory cells. J. Immunol. 179 (3), 1893–1900. 10.4049/jimmunol.179.3.1893 17641056

[B4] Bindea G. Mlecnik B. Tosolini M. Kirilovsky A. Waldner M. Obenauf A. C. (2013). Spatiotemporal dynamics of intratumoral immune cells reveal the immune landscape in human cancer. Immunity 39 (4), 782–795. 10.1016/j.immuni.2013.10.003 24138885

[B5] Bray F. Laversanne M. Sung H. Ferlay J. Siegel R. L. Soerjomataram I. (2024). Global cancer statistics 2022: GLOBOCAN estimates of incidence and mortality worldwide for 36 cancers in 185 countries. CA Cancer J. Clin. 74 (3), 229–263. 10.3322/caac.21834 38572751

[B6] Brueffer C. Vallon-Christersson J. Grabau D. Ehinger A. Hakkinen J. Hegardt C. (2018). Clinical value of RNA sequencing-based classifiers for prediction of the five conventional breast cancer biomarkers: a report from the population-based multicenter Sweden cancerome analysis network-breast initiative. JCO Precis. Oncol. 2, 1–18. 10.1200/PO.17.00135 32913985 PMC7446376

[B7] Charoentong P. Finotello F. Angelova M. Mayer C. Efremova M. Rieder D. (2017). Pan-cancer immunogenomic analyses reveal genotype-immunophenotype relationships and predictors of response to checkpoint blockade. Cell Rep. 18 (1), 248–262. 10.1016/j.celrep.2016.12.019 28052254

[B8] Chen A. Kroehling L. Ennis C. S. Denis G. V. Monti S. (2026). A highly resolved integrated single-cell atlas of human breast cancers. NAR Genom Bioinform 8 (1), lqaf217. 10.1093/nargab/lqaf217 41640877 PMC12867518

[B9] Cichon M. A. Pfisterer K. Leitner J. Wagner L. Staud C. Steinberger P. (2022). Interoperability of RTN1A in dendrite dynamics and immune functions in human langerhans cells. Elife 11, e80578. 10.7554/eLife.80578 36223176 PMC9555864

[B10] Fan Y. Xiao W. Li Z. Li X. Chuang P. Y. Jim B. (2015). RTN1 mediates progression of kidney disease by inducing ER stress. Nat. Commun. 6, 7841. 10.1038/ncomms8841 26227493 PMC4532799

[B11] Fazi B. Melino S. De Rubeis S. Bagni C. Paci M. Piacentini M. (2009). Acetylation of RTN-1C regulates the induction of ER stress by the inhibition of HDAC activity in neuroectodermal tumors. Oncogene 28 (43), 3814–3824. 10.1038/onc.2009.233 19668229

[B12] Fish L. Zhang S. Yu J. X. Culbertson B. Zhou A. Y. Goga A. (2018). Cancer cells exploit an orphan RNA to drive metastatic progression. Nat. Med. 24 (11), 1743–1751. 10.1038/s41591-018-0230-4 30397354 PMC6223318

[B13] Gao Q. Cui Y. Shen Y. Li Y. Gao X. Xi Y. (2019). Identifying mutually exclusive gene sets with prognostic value and novel potential driver genes in patients with glioblastoma. Biomed. Res. Int. 2019, 4860367. 10.1155/2019/4860367 31815141 PMC6878817

[B14] Garcia-Recio S. Hinoue T. Wheeler G. L. Kelly B. J. Garrido-Castro A. C. Pascual T. (2023). Multiomics in primary and metastatic breast tumors from the AURORA US network finds microenvironment and epigenetic drivers of metastasis. Nat. Cancer 4 (1), 128–147. 10.1038/s43018-022-00491-x 36585450 PMC9886551

[B15] Gerratana L. Fanotto V. Bonotto M. Bolzonello S. Minisini A. M. Fasola G. (2015). Pattern of metastasis and outcome in patients with breast cancer. Clin. Exp. Metastasis 32 (2), 125–133. 10.1007/s10585-015-9697-2 25630269

[B16] Gibbs C. So J. Y. Ahad A. Michalowski A. M. Son D. S. Li Y. (2022). CXCL14 attenuates triple-negative breast cancer progression by regulating immune profiles of the tumor microenvironment in a T cell-dependent manner. Int. J. Mol. Sci. 23 (16), 9314. 10.3390/ijms23169314 36012586 PMC9409254

[B17] Gu X. L. Ou Z. L. Lin F. J. Yang X. L. Luo J. M. Shen Z. Z. (2012). Expression of CXCL14 and its anticancer role in breast cancer. Breast Cancer Res. Treat. 135 (3), 725–735. 10.1007/s10549-012-2206-2 22910931

[B18] Han H. Cho J. W. Lee S. Yun A. Kim H. Bae D. (2018). TRRUST v2: an expanded reference database of human and mouse transcriptional regulatory interactions. Nucleic Acids Res. 46 (D1), D380–D386. 10.1093/nar/gkx1013 29087512 PMC5753191

[B19] Ikoma T. Ozawa S. Suzuki K. Kondo T. Maehata Y. Lee M. C. (2012). Calcium-calmodulin signaling induced by epithelial cell differentiation upregulates BRAK/CXCL14 expression *via* the binding of SP1 to the BRAK promoter region. Biochem. Biophys. Res. Commun. 420 (2), 217–222. 10.1016/j.bbrc.2012.01.157 22382027

[B20] Ingebriktsen L. M. Humlevik R. O. C. Svanoe A. A. Saele A. K. M. Winge I. Toska K. (2024). Elevated expression of Aurora-A/AURKA in breast cancer associates with younger age and aggressive features. Breast Cancer Res. 26 (1), 126. 10.1186/s13058-024-01882-x 39198859 PMC11360479

[B21] Jiang P. Gu S. Pan D. Fu J. Sahu A. Hu X. (2018). Signatures of T cell dysfunction and exclusion predict cancer immunotherapy response. Nat. Med. 24 (10), 1550–1558. 10.1038/s41591-018-0136-1 30127393 PMC6487502

[B22] Jiang M. Li X. Xie K. (2025). Pannexin channels in inflammation and tumorigenesis. Front. Cell Dev. Biol. 13, 1647765. 10.3389/fcell.2025.1647765 40909173 PMC12405282

[B23] Khotskaya Y. B. Goverdhan A. Shen J. Ponz-Sarvise M. Chang S. S. Hsu M. C. (2014). S6K1 promotes invasiveness of breast cancer cells in a model of metastasis of triple-negative breast cancer. Am. J. Transl. Res. 6 (4), 361–376. 25075253 PMC4113498

[B24] Kim S. K. Park H. J. Hong I. K. Chung J. H. Eun Y. G. (2013). A missense polymorphism (rs11466653, Met326Thr) of toll-like receptor 10 (TLR10) is associated with tumor size of papillary thyroid carcinoma in the Korean population. Endocrine 43 (1), 161–169. 10.1007/s12020-012-9783-z 23124277

[B25] Li T. Fu J. Zeng Z. Cohen D. Li J. Chen Q. (2020). TIMER2.0 for analysis of tumor-infiltrating immune cells. Nucleic Acids Res. 48 (W1), W509–W514. 10.1093/nar/gkaa407 32442275 PMC7319575

[B26] Li C. Liu H. Wei R. Liu Z. Chen H. Guan X. (2021). LncRNA EGOT/miR-211-5p affected radiosensitivity of rectal cancer by competitively regulating ErbB4. Onco Targets Ther. 14, 2867–2878. 10.2147/OTT.S256989 33953571 PMC8091867

[B27] Liu Y. M. Ge J. Y. Chen Y. F. Liu T. Chen L. Liu C. C. (2023). Combined single-cell and spatial transcriptomics reveal the metabolic evolvement of breast cancer during early dissemination. Adv. Sci. (Weinh) 10 (6), e2205395. 10.1002/advs.202205395 36594618 PMC9951304

[B28] Ma Z. Wang Y. Wang W. Sun W. Wang R. Ding X. (2026). Lipid oxidation reprogramming in cancer-associated fibroblasts enhances CD8(+) T cell cytotoxicity and therapeutic response. Cancer Cell 44, 743–759.e8. 10.1016/j.ccell.2026.01.012 41687608

[B29] Montaudon E. Nikitorowicz-Buniak J. Sourd L. Morisset L. El Botty R. Huguet L. (2020). PLK1 inhibition exhibits strong anti-tumoral activity in CCND1-driven breast cancer metastases with acquired palbociclib resistance. Nat. Commun. 11 (1), 4053. 10.1038/s41467-020-17697-1 32792481 PMC7426966

[B30] Oosting M. Cheng S. C. Bolscher J. M. Vestering-Stenger R. Plantinga T. S. Verschueren I. C. (2014). Human TLR10 is an anti-inflammatory pattern-recognition receptor. Proc. Natl. Acad. Sci. U. S. A. 111 (42), E4478–E4484. 10.1073/pnas.1410293111 25288745 PMC4210319

[B31] Park B. K. Zhang H. Zeng Q. Dai J. Keller E. T. Giordano T. (2007). NF-kappaB in breast cancer cells promotes osteolytic bone metastasis by inducing osteoclastogenesis *via* GM-CSF. Nat. Med. 13 (1), 62–69. 10.1038/nm1519 17159986

[B32] Park M. Kim D. Ko S. Kim A. Mo K. Yoon H. (2022). Breast cancer metastasis: mechanisms and therapeutic implications. Int. J. Mol. Sci. 23 (12), 6806. 10.3390/ijms23126806 35743249 PMC9224686

[B33] Program C. Z. I. C. S. Abdulla S. Aevermann B. Assis P. Badajoz S. Bell S. M. (2025). CZ CELLxGENE discover: a single-cell data platform for scalable exploration, analysis and modeling of aggregated data. Nucleic Acids Res. 53 (D1), D886–D900. 10.1093/nar/gkae1142 39607691 PMC11701654

[B34] Pusztai L. Yau C. Wolf D. M. Han H. S. Du L. Wallace A. M. (2021). Durvalumab with olaparib and paclitaxel for high-risk HER2-negative stage II/III breast cancer: results from the adaptively randomized I-SPY2 trial. Cancer Cell 39 (7), 989–998 e985. 10.1016/j.ccell.2021.05.009 34143979 PMC11064785

[B35] Qiu S. Chen G. Peng J. Liu J. Chen J. Wang J. (2020). LncRNA EGOT decreases breast cancer cell viability and migration *via* inactivation of the hedgehog pathway. FEBS Open Bio 10 (5), 817–826. 10.1002/2211-5463.12833 32150666 PMC7193175

[B36] Ridnour L. A. Cheng R. Y. Switzer C. H. Heinecke J. L. Ambs S. Glynn S. (2013). Molecular pathways: toll-like receptors in the tumor microenvironment--poor prognosis or new therapeutic opportunity. Clin. Cancer Res. 19 (6), 1340–1346. 10.1158/1078-0432.CCR-12-0408 23271799 PMC6314173

[B37] Shao H. Varamini P. (2022). Breast cancer bone metastasis: a narrative review of emerging targeted drug delivery systems. Cells 11 (3), 388. 10.3390/cells11030388 35159207 PMC8833898

[B38] Shen W. Song Z. Zhong X. Huang M. Shen D. Gao P. (2022). Sangerbox: a comprehensive, interaction-friendly clinical bioinformatics analysis platform. Imeta 1 (3), e36. 10.1002/imt2.36 38868713 PMC10989974

[B39] Simon N. Friedman J. Hastie T. Tibshirani R. (2011). Regularization paths for cox's proportional hazards model *via* coordinate descent. J. Stat. Softw. 39 (5), 1–13. 10.18637/jss.v039.i05 27065756 PMC4824408

[B40] Stevens V. L. Hsing A. W. Talbot J. T. Zheng S. L. Sun J. Chen J. (2008). Genetic variation in the toll-like receptor gene cluster (TLR10-TLR1-TLR6) and prostate cancer risk. Int. J. Cancer 123 (11), 2644–2650. 10.1002/ijc.23826 18752252

[B41] Sun W. Duan T. Ye P. Chen K. Zhang G. Lai M. (2018). TSVdb: a web-tool for TCGA splicing variants analysis. BMC Genomics 19 (1), 405. 10.1186/s12864-018-4775-x 29843604 PMC5975414

[B42] Tan L. Xiang J. Lu Y. Zhong X. (2025). Uncovering GSTK1 and PTGER3 as biomarkers for breast cancer prognosis through comprehensive analyses. Discov. Oncol. 17 (1), 163. 10.1007/s12672-025-04329-7 41432840 PMC12847519

[B43] Verma R. Jung J. H. Kim J. Y. (2014). 1,25-Dihydroxyvitamin D3 up-regulates TLR10 while down-regulating TLR2, 4, and 5 in human monocyte THP-1. J. Steroid Biochem. Mol. Biol. 141, 1–6. 10.1016/j.jsbmb.2013.12.012 24373795

[B44] Wang M. Wei Z. Wang S. Feng W. Shang L. Sun X. (2023a). Long non-coding RNA EGOT is associated with (131)iodine sensitivity and contributes to thyroid cancer progression by targeting miR-641/PTEN axis. Aging (Albany NY) 15 (22), 13542–13557. 10.18632/aging.205284 38006396 PMC10713430

[B45] Wang M. Wu Y. Li X. Dai M. Li S. (2023b). IGJ suppresses breast cancer growth and metastasis by inhibiting EMT *via* the NF-kappaB signaling pathway. Int. J. Oncol. 63 (3), 105. 10.3892/ijo.2023.5553 37539706 PMC10552693

[B46] Wherry E. J. Kurachi M. (2015). Molecular and cellular insights into T cell exhaustion. Nat. Rev. Immunol. 15 (8), 486–499. 10.1038/nri3862 26205583 PMC4889009

[B47] Xu Z. Wang W. Yang T. Li L. Ma X. Chen J. (2024). STOmicsDB: a comprehensive database for spatial transcriptomics data sharing, analysis and visualization. Nucleic Acids Res. 52 (D1), D1053–D1061. 10.1093/nar/gkad933 37953328 PMC10767841

[B48] Yang H. Wang B. Wang T. Xu L. He C. Wen H. (2014). Toll-like receptor 4 prompts human breast cancer cells invasiveness *via* lipopolysaccharide stimulation and is overexpressed in patients with lymph node metastasis. PLoS One 9 (10), e109980. 10.1371/journal.pone.0109980 25299052 PMC4192367

[B49] Zhou X. X. Jia W. H. Shen G. P. Qin H. D. Yu X. J. Chen L. Z. (2006). Sequence variants in toll-like receptor 10 are associated with nasopharyngeal carcinoma risk. Cancer Epidemiol. Biomarkers Prev. 15 (5), 862–866. 10.1158/1055-9965.EPI-05-0874 16702361

