# Supplementary material for: Identification and validation of a seven-gene metastasis-associated prognostic model in breast cancer
Source: Front Genet. 2026 May 11;17:1770418. doi: 10.3389/fgene.2026.1770418 (PMC13198929; doi:10.3389/fgene.2026.1770418)
Supplement: Supplementary file 1 [file Table1.docx]

**Supplementary Table S1. qPCR primers.**

| **Target** | **Primer** | **Sequence (5’-> 3’)** |
| --- | --- | --- |
| 18SrRNA | Forward | AGTCCCTGCCCTTTGTACACA |
|  | Reverse | CGATCCGAGGGCCTCACTA |
| IGJ | Forward | CCAGGATCATCCGTTCTTCCGA |
|  | Reverse | CAAATCTGGTTCTCAATGGTGAGG |
| CXCL14 | Forward | CGCTACAGCGACGTGAAGAA |
|  | Reverse | GTTCCAGGCGTTGTACCAC |
| RTN1 | Forward | CAGTCCCCACTGTCACTGTC |
| isoform A | Reverse | GGTGATCAGCTCATCCTCGG |
| RTN1 | Forward | CACTGCCGATTCCACCAAGA |
| isoform C | Reverse | TCCCGCCAATACAACAGGTC |
| EGOT | Forward | CATCTCAGTCCAGTGCCCAG |
|  | Reverse | GCACCCTGTTCATAAGCCCT |
| PTGER3 | Forward | CGCCTCAACCACTCCTACAC |
|  | Reverse | GACACCGATCCGCAATCCTC |
| PANX2 | Forward | CCTCACCATCCTGAGCCGAAAC |
|  | Reverse | GACGGTGCCATCCCTCAAAACT |
| TLR10 | Forward | AGGTTTGAGTGGGGCAAAAAT |
|  | Reverse | CCATCACGCAAAAGAACCCAG |

**Supplementary Table S2.** **Calibration performance of the prognostic signature.**

| **Cohort** | **SCAN-B training** | **SCAN-B validation** | **TCGA-BRCA** |
| --- | --- | --- | --- |
| **Sample size** | 3037 | 340 | 1081 |
| **Time point (days)** | 1095 | 1095 | 1095 |
| **Calibration slope** | 1.158495 | 1.509184 | 0.724172 |
| **Observed risk** | 0.057400 | 0.059545 | 0.099346 |
| **Expected risk** | 0.057549 | 0.059493 | 0.102633 |
| **O/E ratio** | 0.997408 | 1.000870 | 0.967975 |
| **Calibration-in-the-large** | -0.002595 | 0.000870 | -0.03255 |

**Supplementary Table S3.** **Clinical characteristics of low- and high-risk groups in SCAN-B training set.**

| **Risk score** | **total(n=3069)** | **low(n=1535)** | **high(n=1534)** | **p-value** |
| --- | --- | --- | --- | --- |
| **Age** | 62.76±13.11 | 61.19±12.18 | 64.33±13.80 | **2.81E-11** |
| **Tumor size** | 19.98±12.43 | 18.25±11.58 | 21.73±13.00 | **9.69E-15** |
| **Lymph node status** |  |  |  | 0.12 |
| Negative | 1878(63.17%) | 963(32.39%) | 915(30.78%) |  |
| Positive | 1095(36.83%) | 528(17.76%) | 567(19.07%) |  |
| **ER status** |  |  |  | **5.50E-35** |
| Negative | 224(7.80%) | 28(0.98%) | 196(6.83%) |  |
| Positive | 2646(92.20%) | 1472(51.29%) | 1174(40.91%) |  |
| **PGR status** |  |  |  | **3.50E-35** |
| Negative | 362(13.22%) | 80(2.92%) | 282(10.30%) |  |
| Positive | 2377(86.78%) | 1358(49.58%) | 1019(37.20%) |  |
| **HER2 status** |  |  |  | **4.90E-12** |
| Negative | 2572(86.77%) | 1345(45.38%) | 1227(41.40%) |  |
| Positive | 392(13.23%) | 131(4.42%) | 261(8.81%) |  |
| **Ki67 status** |  |  |  | **8.50E-29** |
| Negative | 574(41.38%) | 389(28.05%) | 185(13.34%) |  |
| Positive | 813(58.62%) | 303(21.85%) | 510(36.77%) |  |
| **NHG** |  |  |  | **2.70E-88** |
| G1 | 454(15.09%) | 352(11.47%) | 102(3.32%) |  |
| G2 | 1439(47.84%) | 848(27.63%) | 591(19.26%) |  |
| G3 | 1115(37.07%) | 310(10.10%) | 805(26.23%) |  |
| **PAM50** |  |  |  | **2.40E-174** |
| Basal-like | 325(10.59%) | 46(1.50%) | 279(9.09%) |  |
| HER2-enriched | 307(10.00%) | 51(1.66%) | 256(8.34%) |  |
| LumA | 1540(50.18%) | 1102(35.91%) | 438(14.27%) |  |
| LumB | 695(22.65%) | 180(5.87%) | 515(16.78%) |  |
| Normal | 202(6.58%) | 156(5.08%) | 46(1.50%) |  |

**Supplementary Table S4. Clinical characteristics of low- and high-risk groups in TCGA-BRCA.**

| **Risk score** | **total(n=1081)** | **low(n=541)** | **high(n=540)** | **p-value** |
| --- | --- | --- | --- | --- |
| **Age** | 58.30±13.16 | 57.16±13.14 | 59.44±13.09 | **0.0043** |
| **Initial weight** | 294.00±267.27 | 315.45±259.09 | 304.75±263.28 | 0.23 |
| **Metastasis** |  |  |  | 0.21 |
| M0 | 757(98.18%) | 538(49.77%) | 531(49.12%) |  |
| M1 | 14(1.82%) | 3(0.28%) | 9(0.83%) |  |
| **Node** |  |  |  | 0.21 |
| N0 | 375(48.26%) | 185(23.81%) | 190(24.45%) |  |
| N1 | 261(33.59%) | 136(17.50%) | 125(16.09%) |  |
| N2 | 99(12.74%) | 40(5.15%) | 59(7.59%) |  |
| N3 | 42(5.41%) | 18(2.32%) | 24(3.09%) |  |
| **Tumor** |  |  |  | **1.80E-08** |
| T1 | 209(26.90%) | 139(17.89%) | 70(9.01%) |  |
| T2 | 461(59.33%) | 201(25.87%) | 260(33.46%) |  |
| T3 | 75(9.65%) | 32(4.12%) | 43(5.53%) |  |
| T4 | 29(3.73%) | 6(0.77%) | 23(2.96%) |  |
| TX | 3(0.39%) | 1(0.13%) | 2(0.26%) |  |
| **Stage** |  |  |  | **7.00E-04** |
| I | 181(16.87%) | 116(10.81%) | 65(6.06%) |  |
| II | 612(57.04%) | 290(27.03%) | 322(30.01%) |  |
| III | 246(22.93%) | 119(11.09%) | 127(11.84%) |  |
| IV | 20(1.86%) | 7(0.65%) | 13(1.21%) |  |
| X | 14(1.30%) | 5(0.47%) | 9(0.84%) |  |
| **PAM50** |  |  |  | **1.10E-40** |
| Basal-like | 139(16.73%) | 26(3.13%) | 113(13.60%) |  |
| HER2-enriched | 64(7.70%) | 15(1.81%) | 49(5.90%) |  |
| LumA | 417(50.18%) | 300(36.10%) | 117(14.08%) |  |
| LumB | 189(22.74%) | 56(6.74%) | 133(16.00%) |  |
| Normal | 22(2.65%) | 18(2.17%) | 4(0.48%) |  |
| **PR Status** |  |  |  | **1.40E-26** |
| Negative | 340(32.88%) | 89(8.61%) | 251(24.27%) |  |
| Positive | 690(66.73%) | 429(41.49%) | 261(25.24%) |  |
| Indeterminate | 4(0.39%) | 3(0.29%) | 1(0.10%) |  |
| **ER Status** |  |  |  | **1.20E-20** |
| Negative | 238(23.00%) | 55(5.31%) | 183(17.68%) |  |
| Positive | 795(76.81%) | 465(44.93%) | 330(31.88%) |  |
| Indeterminate | 2(0.19%) | 1(0.10%) | 1(0.10%) |  |
| **HER2 Status** |  |  |  | **8.60E-04** |
| Negative | 866(82.55%) | 456(43.47%) | 410(39.08%) |  |
| Positive | 158(15.06%) | 57(5.43%) | 101(9.63%) |  |
| Indeterminate | 8(0.76%) | 5(0.48%) | 3(0.29%) |  |
| Equivocal | 17(1.62%) | 11(1.05%) | 10(1.29%) |  |

**Supplementary Table S5.** **Performance evaluation of four prognostic models.**

| **Model name** | **Metric** | **Estimate(95%Cl)** |
| --- | --- | --- |
| Model A (Clinical) | C-index | 0.858(0.821-0.926) |
| Model B (Clinical+PAM50) | C-index | 0.865(0.834-0.934) |
| Model C (Clinical+7-gene) | C-index | 0.867(0.833-0.932) |
| Model D (Clinical+Elastic-net) | C-index | 0.767(0.676-0.852) |
| Model A (Clinical) | AUC(1095) | 0.856(0.794-0.918) |
| Model B (Clinical+PAM50) | AUC(1095) | 0.865(0.806-0.924) |
| Model C (Clinical+7-gene) | AUC(1095) | 0.870(0.815-0.925) |
| Model D (Clinical+Elastic-net) | AUC(1095) | 0.759(0.655-0.862) |
| Model A (Clinical) | AUC(1825) | 0.870(0.806-0.934) |
| Model B (Clinical+PAM50) | AUC(1825) | 0.883(0.823-0.942) |
| Model C (Clinical+7-gene) | AUC(1825) | 0.864(0.796-0.932) |
| Model D (Clinical+Elastic-net) | AUC(1825) | 0.841(0.754-0.928) |

**Supplementary Table S6.** **The top three cell subsets with the highest gene expression (by single-cell sequencing).**

| **Gene** | **Top 1** | **Top 2** | **Top 3** |
| --- | --- | --- | --- |
| IGJ | plasmacytoid dendritic cell | IgG plasma cell | memory B cell |
| CXCL14 | fibroblast | malignant cell | capillary endothelial cell |
| PTGER3 | pericyte | fibroblast | capillary endothelial cell |
| EGOT | malignant cell | cycling stromal cell | T follicular helper cell |
| RTN1 | conventional dendritic cell | cycling macrophage | macrophage |
| TLR10 | memory B cell | conventional dendritic cell | plasmacytoid dendritic cell |
| PANX2 | cycling stromal cell | malignant cell | plasmacytoid dendritic cell |
| 7-gene set | plasmacytoid dendritic cell | IgG plasma cell | fibroblast |

**Supplementary Table S7.** **Performance of the refined prognostic model according to single-cell sequencing data.**

| **Model name** | **Metric** | **Estimate(95%Cl)** |
| --- | --- | --- |
| 7-gene | C-index | 0.683(0.654-0.712) |
| immune + malignant | C-index | 0.666(0.636-0.695) |
| immune + stromal | C-index | 0.669(0.639-0.699) |
| malignant + stromal | C-index | 0.654(0.624-0.685) |
| immune | C-index | 0.630(0.599-0.660) |
| malignant | C-index | 0.618(0.586-0.651) |
| stromal | C-index | 0.639(0.608-0.671) |
| 7-gene | AUC(365) | 0.76(0.67-0.86) |
| immune + malignant | AUC(365) | 0.73(0.63-0.82) |
| immune + stromal | AUC(365) | 0.74(0.63-0.84) |
| malignant + stromal | AUC(365) | 0.77(0.68-0.85) |
| immune | AUC(365) | 0.65(0.53-0.76) |
| malignant | AUC(365) | 0.70(0.59-0.80) |
| stromal | AUC(365) | 0.75 (0.65-0.84) |
| 7-gene | AUC(1095) | 0.72(0.68-0.72) |
| immune + malignant | AUC(1095) | 0.70(0.66-0.73) |
| immune + stromal | AUC(1095) | 0.70(0.66-0.74) |
| malignant + stromal | AUC(1095) | 0.69(0.65-0.73) |
| immune | AUC(1095) | 0.65(0.61-0.69) |
| malignant | AUC(1095) | 0.64(0.60-0.68) |
| stromal | AUC(1095) | 0.67 (0.63-0.71) |
| 7-gene | AUC(1825) | 0.69(0.66-0.72) |
| immune + malignant | AUC(1825) | 0.68(0.64-0.71) |
| immune + stromal | AUC(1825) | 0.68(0.64-0.71) |
| malignant + stromal | AUC(1825) | 0.65(0.61-0.68) |
| immune | AUC(1825) | 0.65(0.62-0.68) |
| malignant | AUC(1825) | 0.61(0.58-0.65) |
| stromal | AUC(1825) | 0.63 (0.60-0.67) |

Immune-related gene: IGJ, RTN1, TLR10

Stromal-related gene: CXCL14, PTGER3, PANX2

Malignant-related gene: EGOT
