# Supplementary material for: Identification and validation of a seven-gene metastasis-associated prognostic model in breast cancer
Source: Front Genet. 2026 May 11;17:1770418. doi: 10.3389/fgene.2026.1770418 (PMC13198929; doi:10.3389/fgene.2026.1770418)

IGJ

PCR product length: 126 bp

Above: MCF-7, lanes 1-4; MDA-MB-468, lanes 5-8; SKBR3, lanes 9-12; DNA ladder, lane 13.

Below: MCF-10A, lanes 1-4; MDA-MB-231, lanes 5-8; DNA ladder, lane 9.


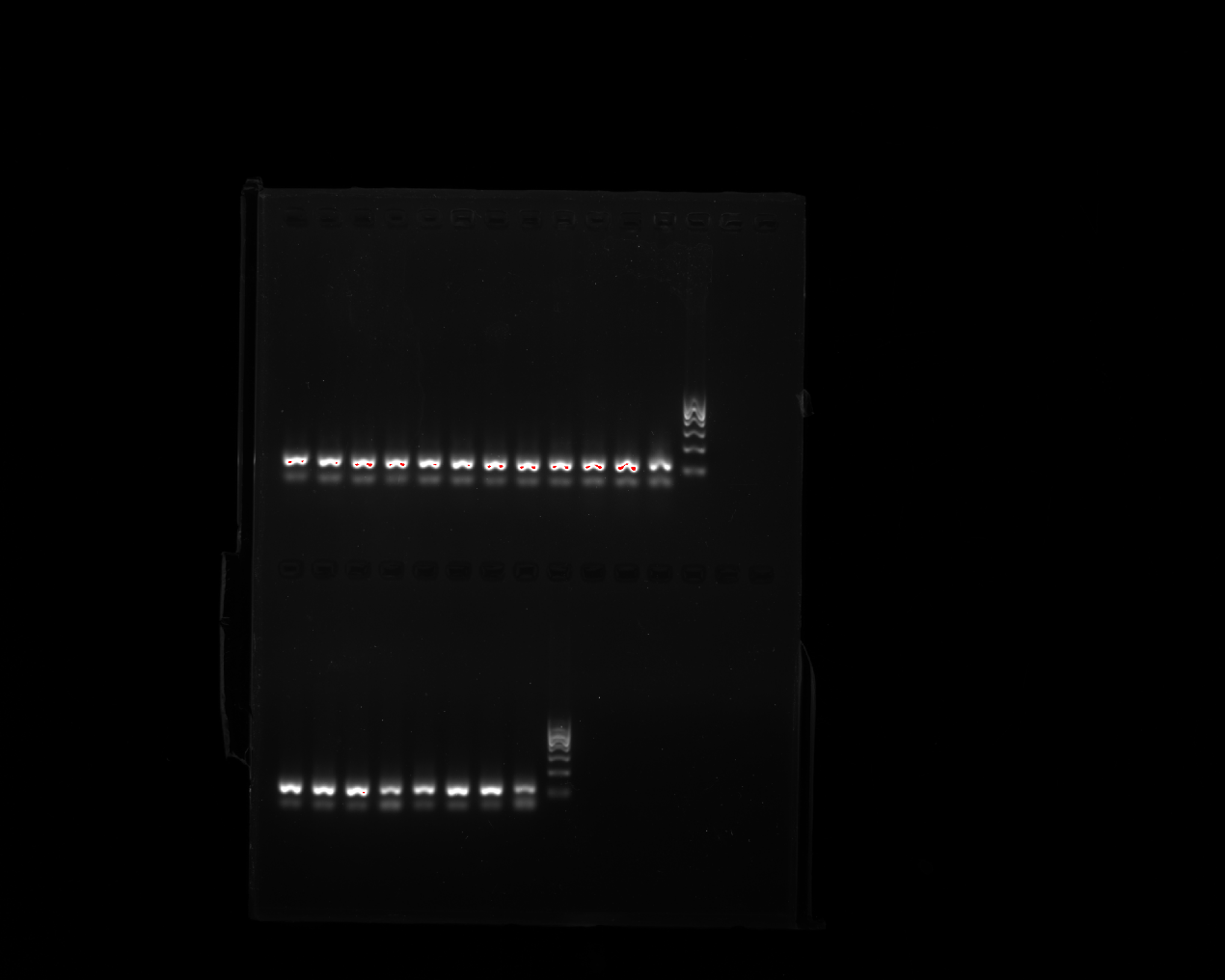


CXCL14

PCR product length: 171 bp

Above: MCF-7, lanes 1-4; MDA-MB-468, lanes 5-8; SKBR3, lanes 9-12; DNA ladder, lane 13.

Below: MCF-10A, lanes 1-4; MDA-MB-231, lanes 5-8; DNA ladder, lane 9.


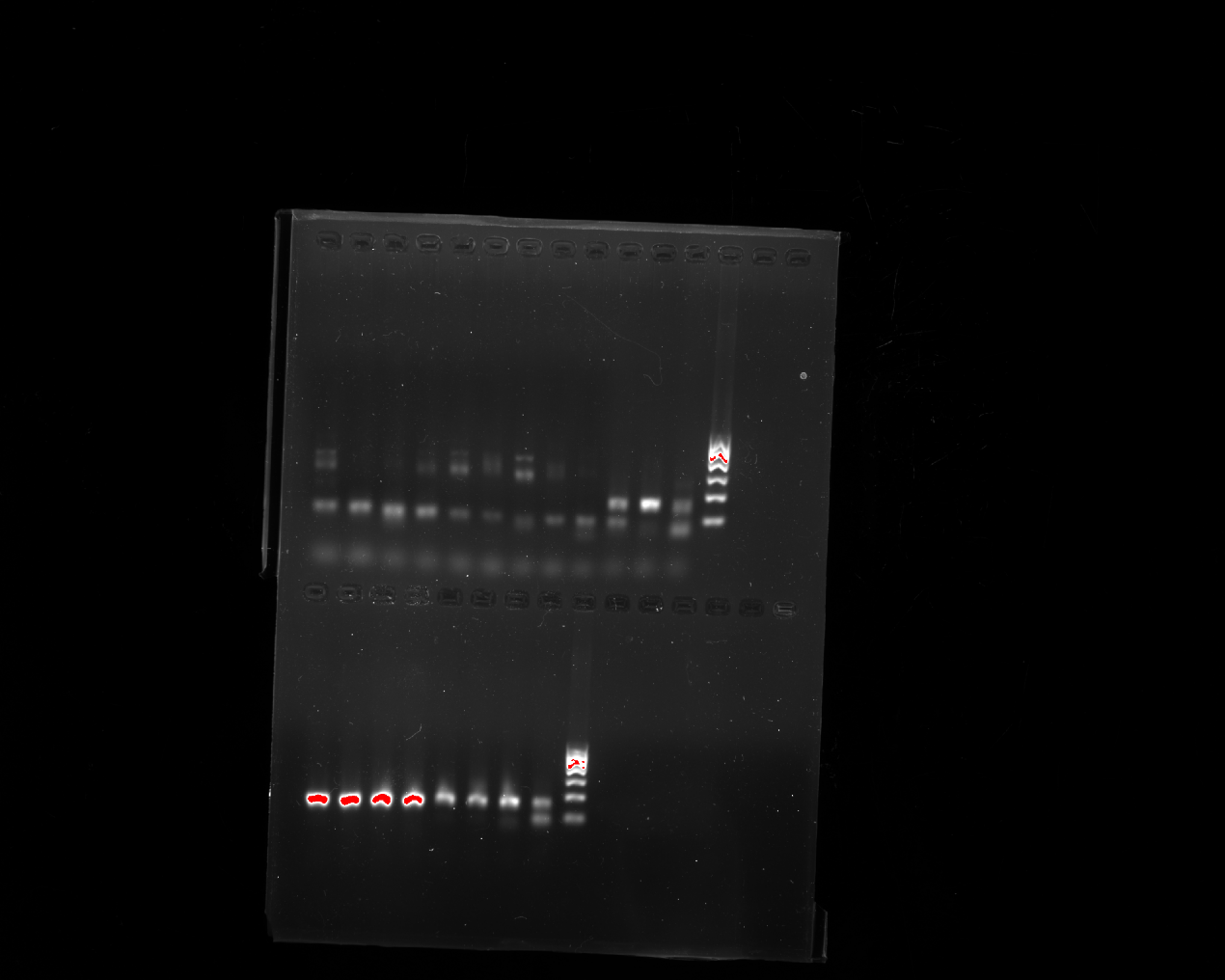


RTN1-A

PCR product length: 134 bp

Above: MCF-7, lanes 1-4; MDA-MB-468, lanes 5-8; SKBR3, lanes 9-12; DNA ladder, lane 13.

Below: MCF-10A, lanes 1-4; MDA-MB-231, lanes 5-8; DNA ladder, lane 9.


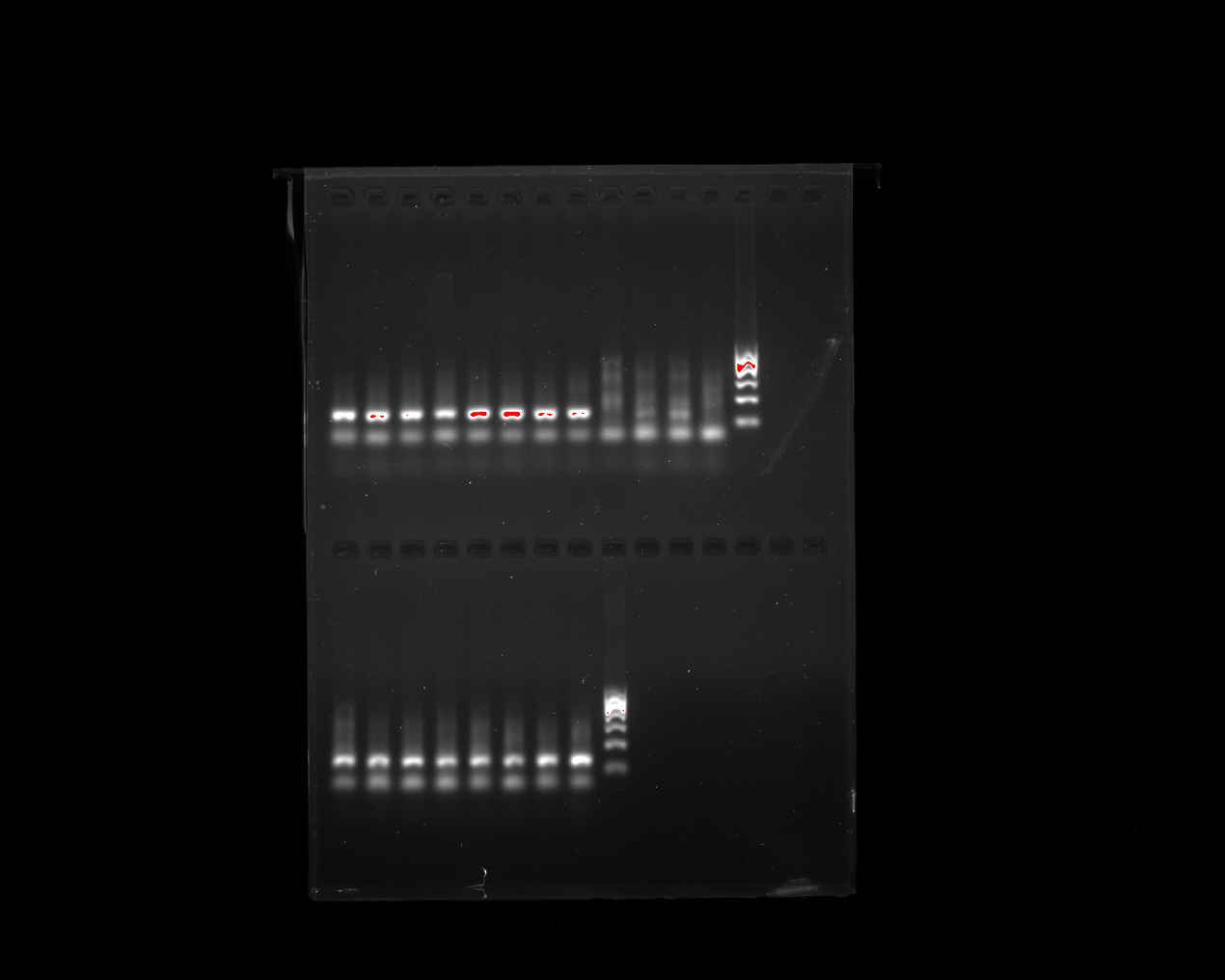


RTN1-C

PCR product length: 78 bp

Above: MCF-7, lanes 1-4; MDA-MB-468, lanes 5-8; SKBR3, lanes 9-12; DNA ladder, lane 13.

Below: MCF-10A, lanes 4-7; MDA-MB-231, lanes 8-11; DNA ladder, lane 12.


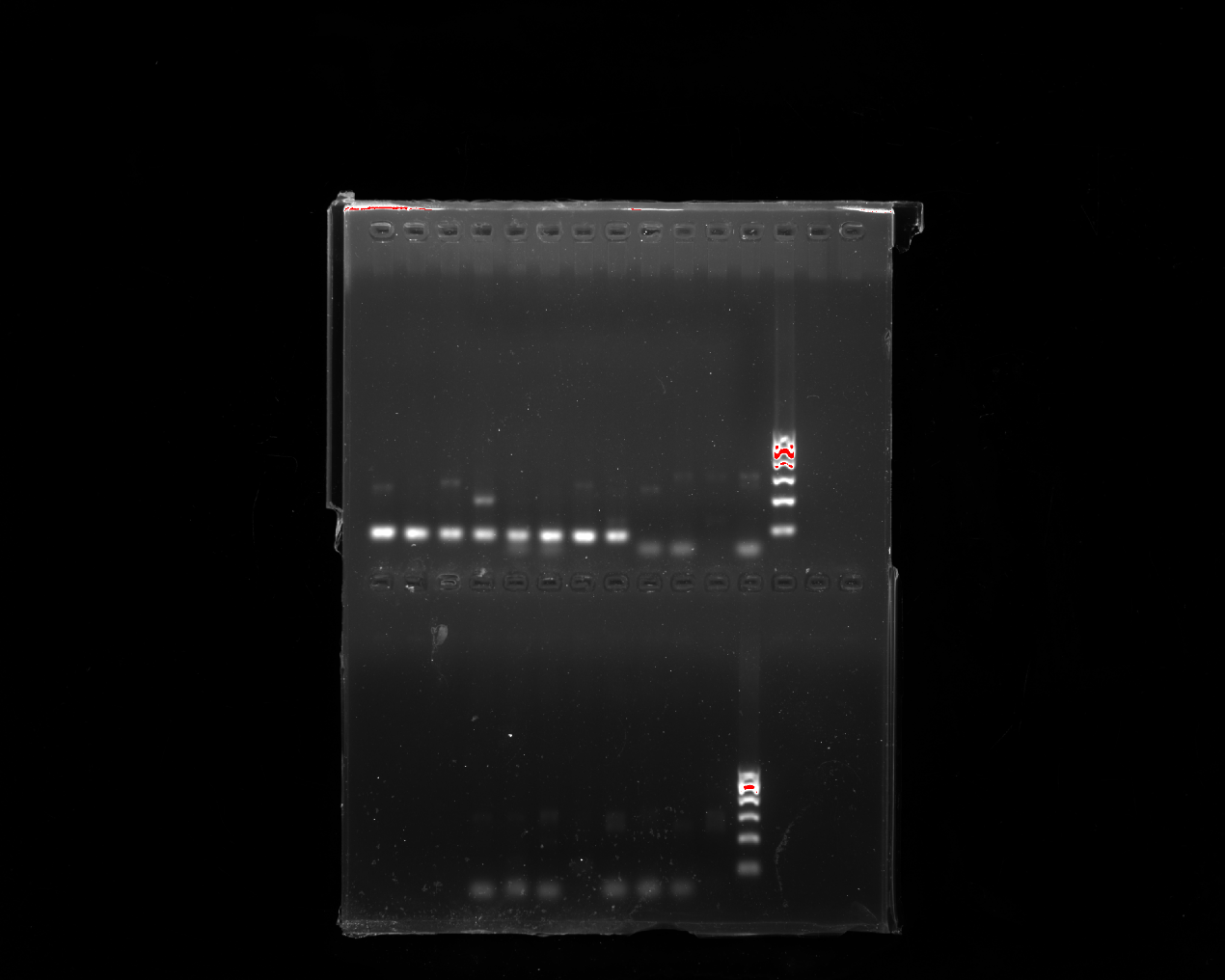


TLR10

PCR product length: 189 bp

Above: MCF-7, lanes 1-4; MDA-MB-468, lanes 5-8; SKBR3, lanes 9-12; DNA ladder, lane 13.

Below: MCF-10A, lanes 1-4; MDA-MB-231, lanes 5-8; DNA ladder, lane 9.


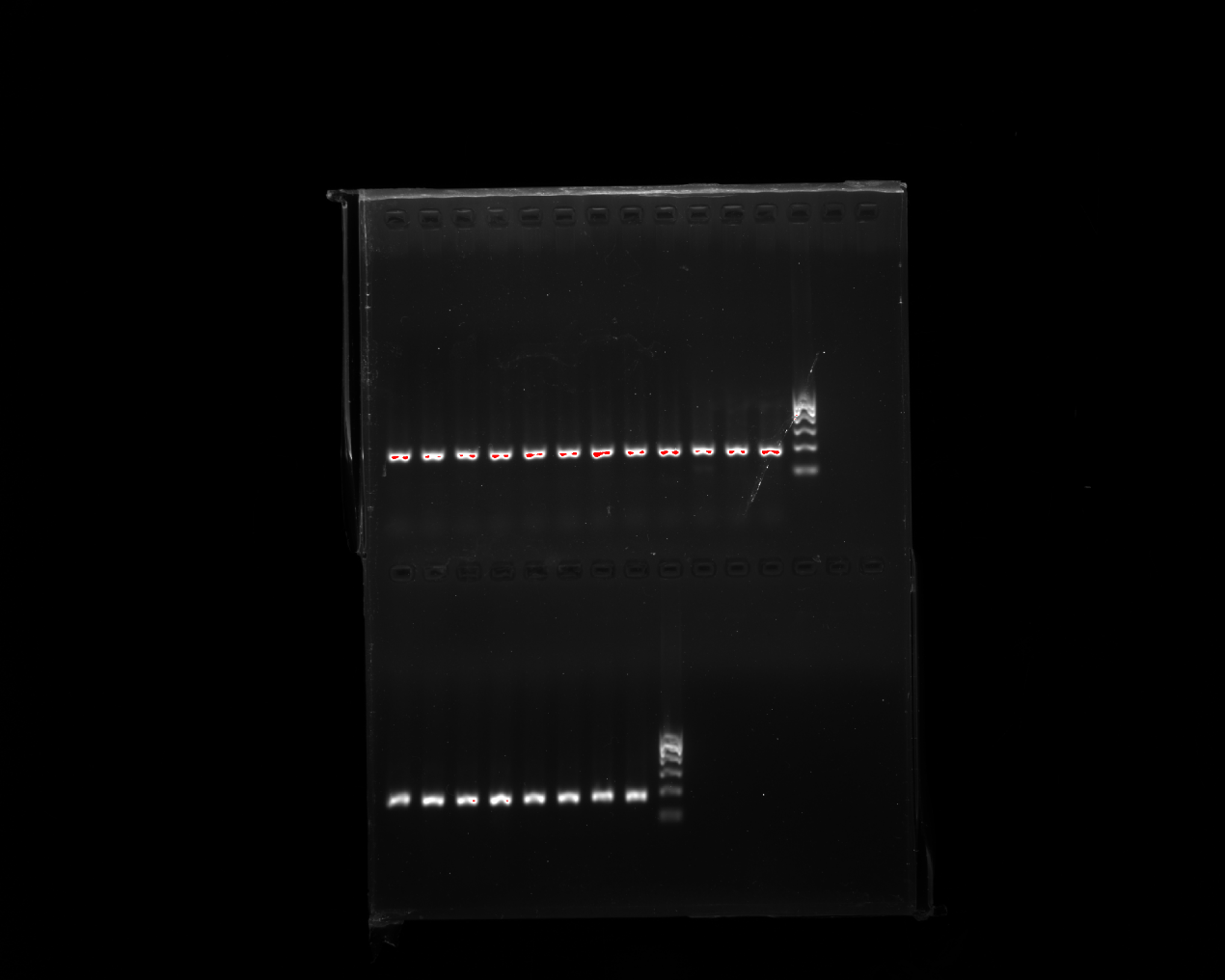


PANX2

PCR product length: 210 bp

Above: MCF-7, lanes 1-4; MDA-MB-468, lanes 5-8; SKBR3, lanes 9-12; DNA ladder, lane 13.

Below: MCF-10A, lanes 1-4; MDA-MB-231, lanes 5-8; DNA ladder, lane 9.


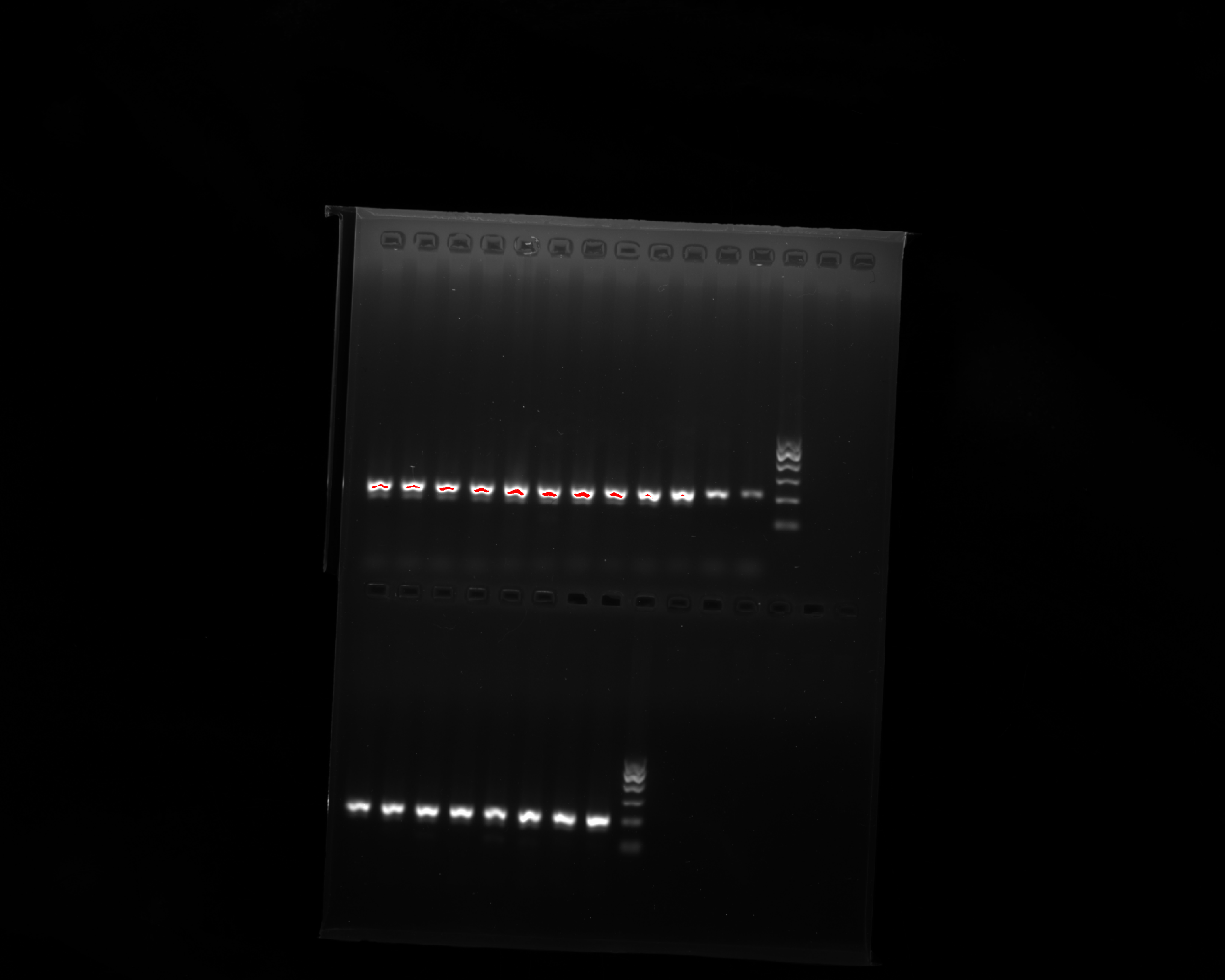


EGOT

PCR product length: 63 bp

Above: MCF-7, lanes 1-4; MDA-MB-468, lanes 5-8; SKBR3, lanes 9-12; DNA ladder, lane 13.

Below: MCF-10A, lanes 1-4; MDA-MB-231, lanes 5-8; DNA ladder, lane 9.


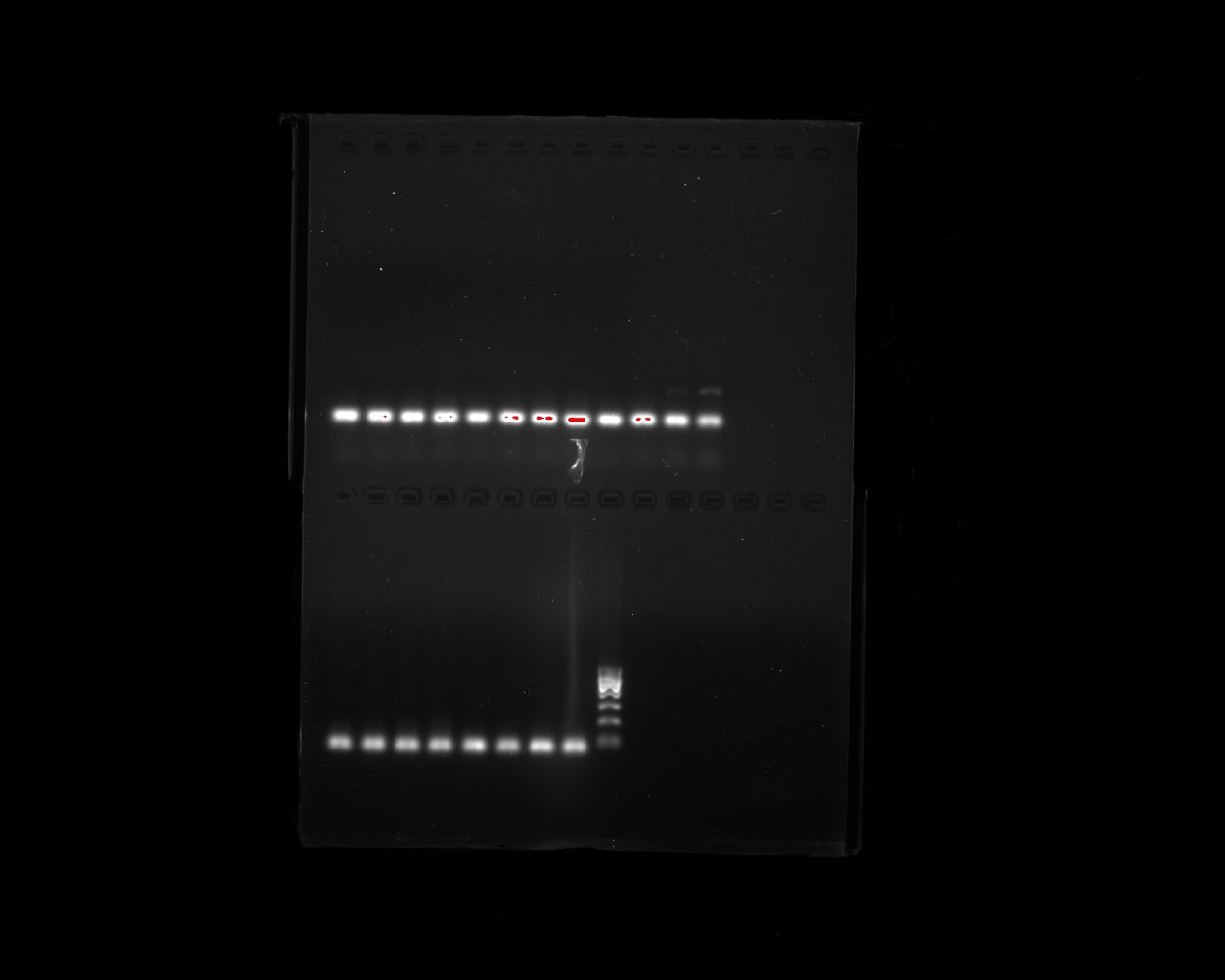


PTGER3

PCR product length: 107 bp

Above: MCF-7, lanes 1-4; MDA-MB-468, lanes 5-8; SKBR3, lanes 9-12; DNA ladder, lane 13.

Below: MCF-10A, lanes 1-4; MDA-MB-231, lanes 5-8; DNA ladder, lane 9.


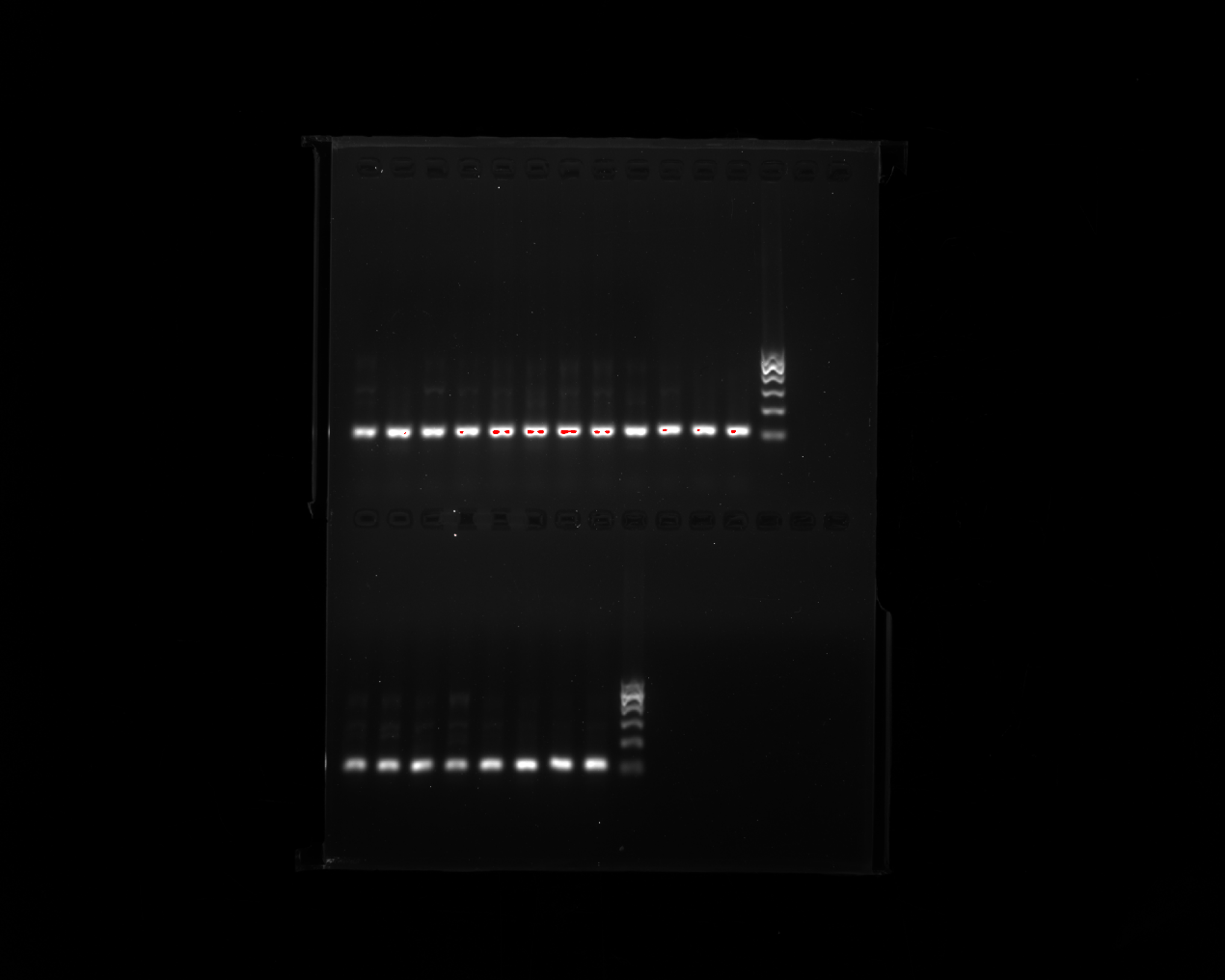

Supplement: Supplementary file 2 [file DataSheet2.docx]
