# Supplementary material for: Identification and validation of a seven-gene metastasis-associated prognostic model in breast cancer
Source: Front Genet. 2026 May 11;17:1770418. doi: 10.3389/fgene.2026.1770418 (PMC13198929; doi:10.3389/fgene.2026.1770418)
Supplement: Supplementary file 3 [file DataSheet1.docx]

**
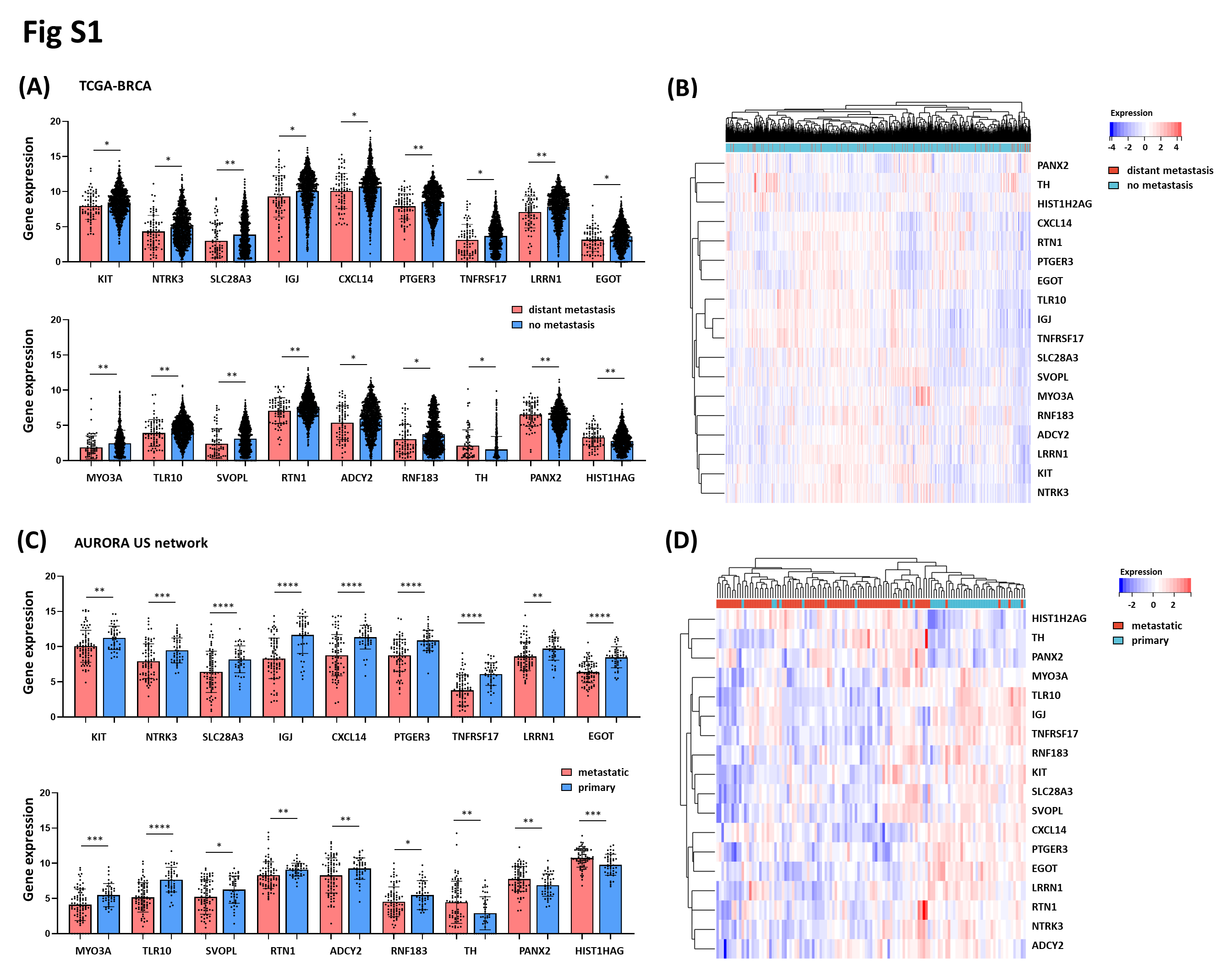
**

**Figure S1.** M-CA-DEGs in BRCA. (A) Expression of M-CA-DEGs between the metastasis and nonmetastasis groups in the TCGA-BRCA cohort. (B) Heatmap and clustering analysis of M-CA-DEGs between the metastasis and nonmetastasis groups in the TCGA-BRCA cohort. (C) Expression of M-CA-DEGs between the metastatic and primary groups in the AURORA US Network. (D) Heatmap and clustering analysis of M-CA-DEGs between the metastatic and primary groups in the AURORA US Network. The data are presented as the means ± SDs. The heatmap and clustering analysis were performed via Euclidean distance and the complete linkage method. P values were calculated via Student’s unpaired t test. *, P < 0.05; **, P < 0.01; ***, P < 0.001; ****, P < 0.0001.


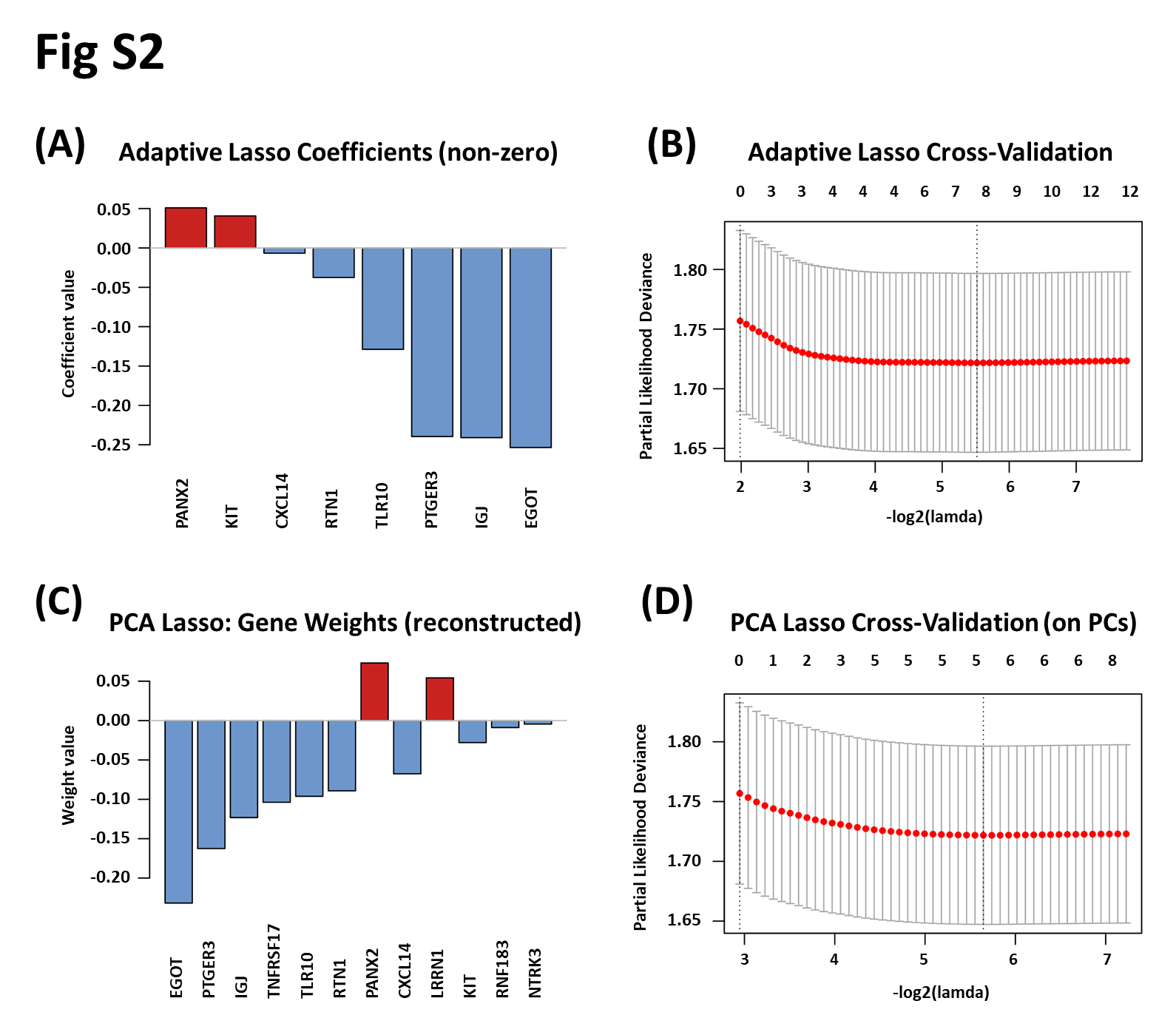


**Figure S2.** Construction of the prognostic model in BRCA. (A) Coefficients from adaptive LASSO regression in the SCAN-B training set. (B) Cross‑validation of the adaptive LASSO model. (C) Gene weights derived from PCA‑weighted LASSO regression. (D) Cross‑validation of the PCA‑weighted LASSO model.


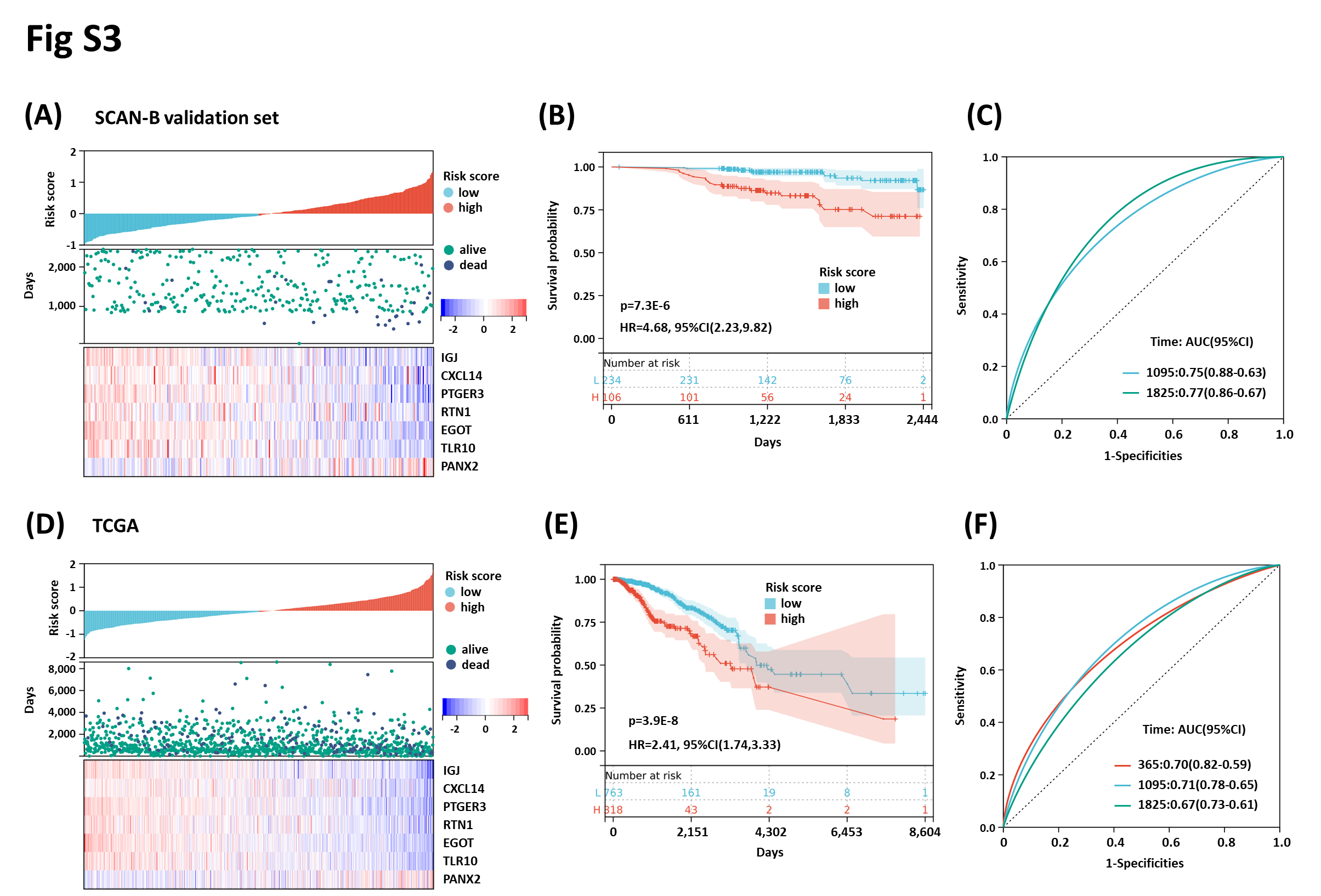


**Figure S3.** Validation of the prognostic model. Heatmap of the risk score and prognostic genes in the SCAN-B validation set (A) and TCGA cohort (D). Kaplan‒Meier curves of the prognostic model in the SCAN-B validation set (B) and TCGA cohort (E). ROC curve of the prognostic model in the SCAN-B validation set (C) and TCGA cohort (F). P values were calculated via the log-rank test.


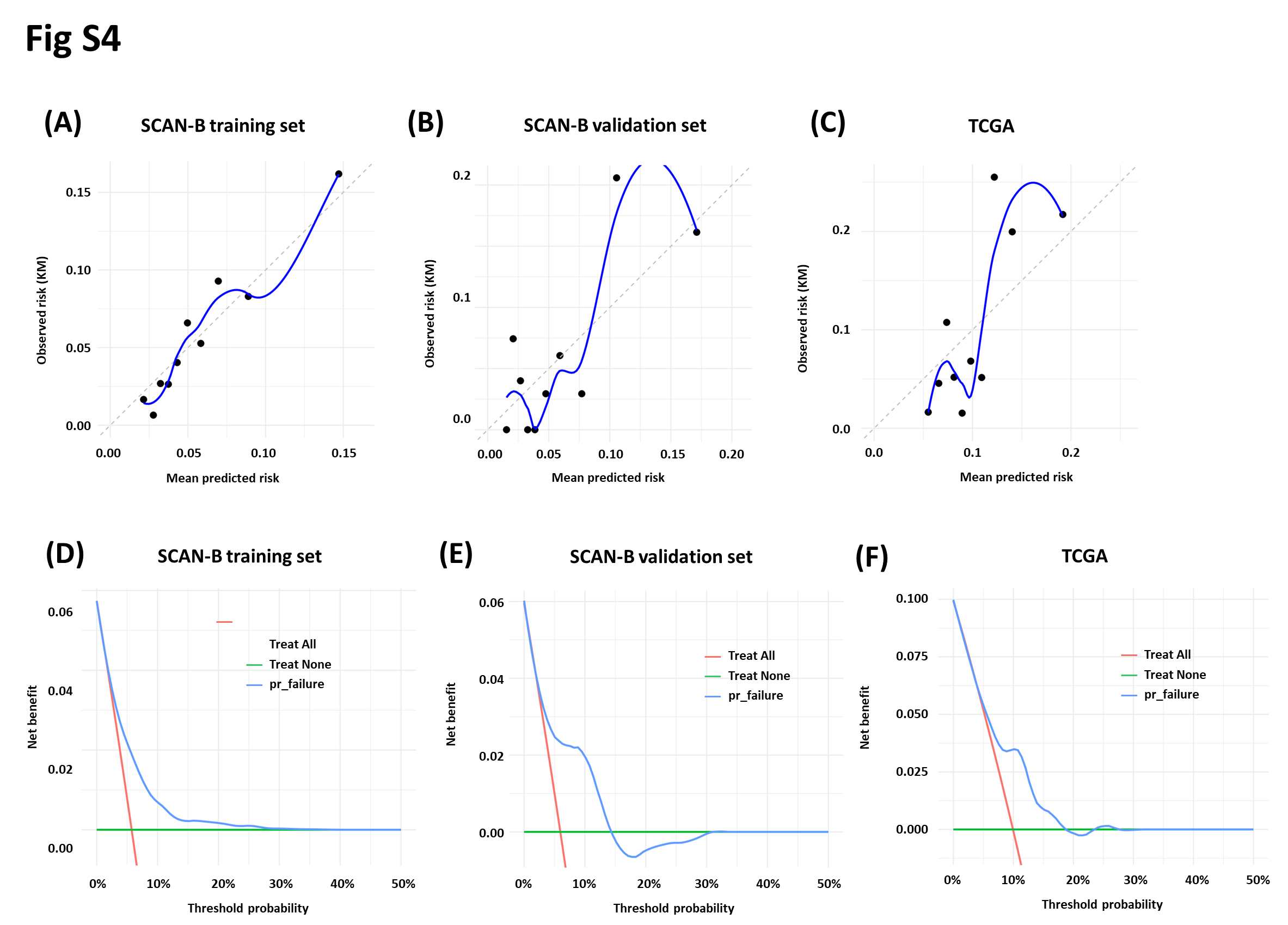


**Figure S4.** Validation of the prognostic model. Calibration analysis of the prognostic model in the SCAN-B training set (A), the SCAN-B validation set (B) and TCGA cohort (C). Decision curve analysis (DCA) of the prognostic model in the SCAN-B training set (D), the SCAN-B validation set (E) and TCGA cohort (F).


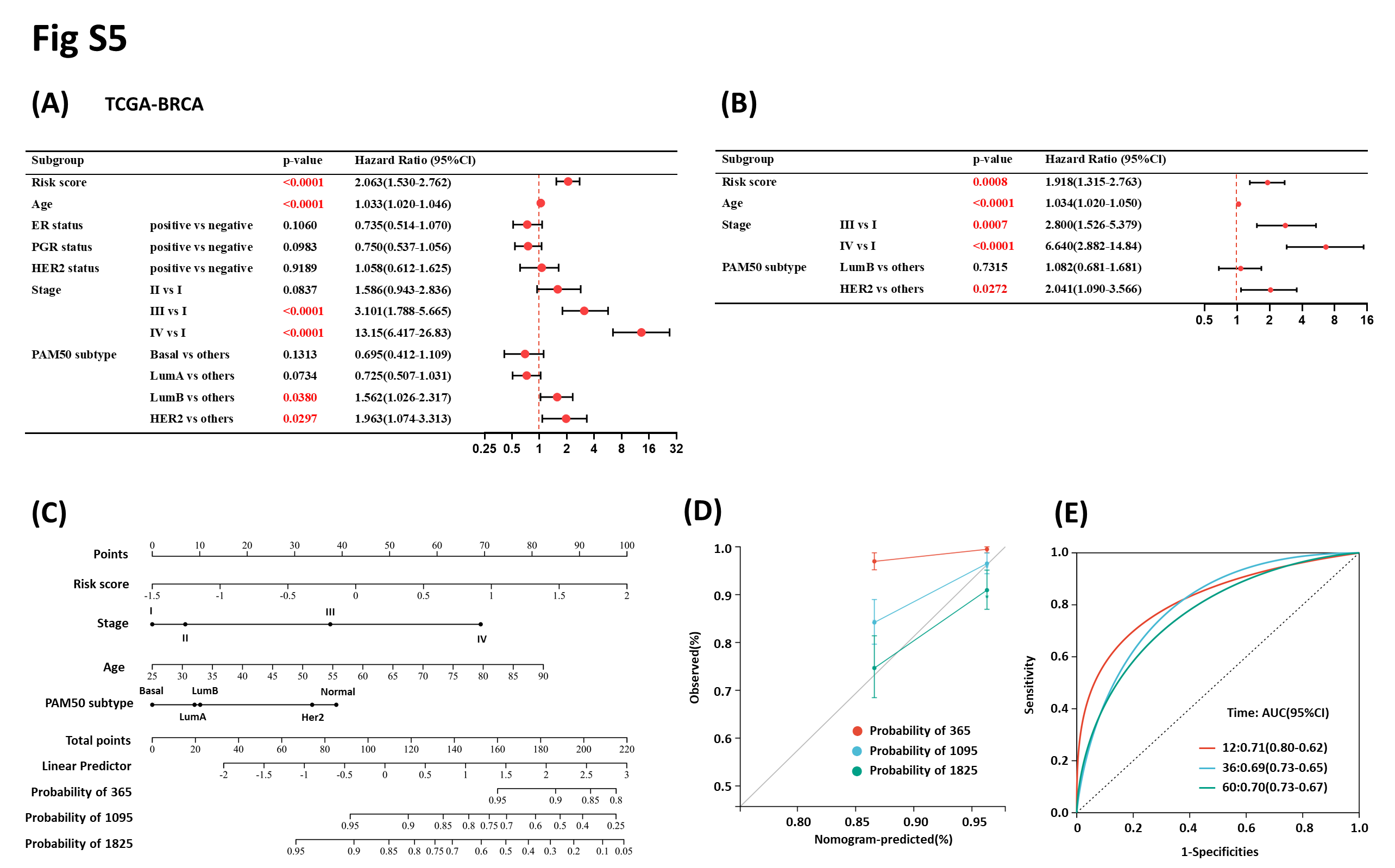


**Figure S5.** Validation of the risk score as an independent prognostic factor. Univariate (A) and multivariate (B) Cox regression analyses of prognostic factors in the TCGA-BRCA cohort. Independent prognostic factors were identified via the nomogram (C) and calibration curves (D). (E) ROC curve of the nomogram. P values were calculated via the log-rank test.


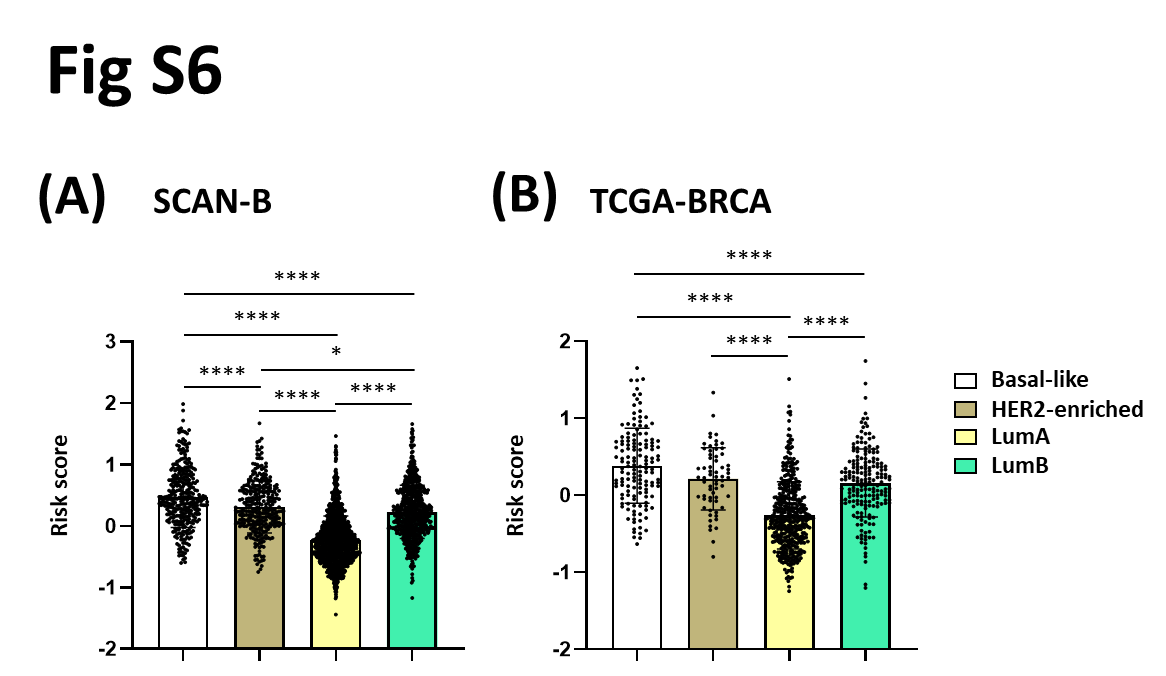


**Figure S6.** Risk score as a prognostic factor in BRCA subtypes. (A) The risk scores of Basal-like (n=325), HER2-enriched (n=307), LumA (n=1540), and LumB (n=695) patients from the SCAN-B cohort. (B) The risk scores of Basal-like (n=139), HER2-enriched (n=64), LumA (n=417), and LumB (n=189) patients from the TCGA-BRCA patients. The data are presented as the means ± SDs. P values were calculated via one-way ANOVA. *, P < 0.05; ****, P < 0.0001.


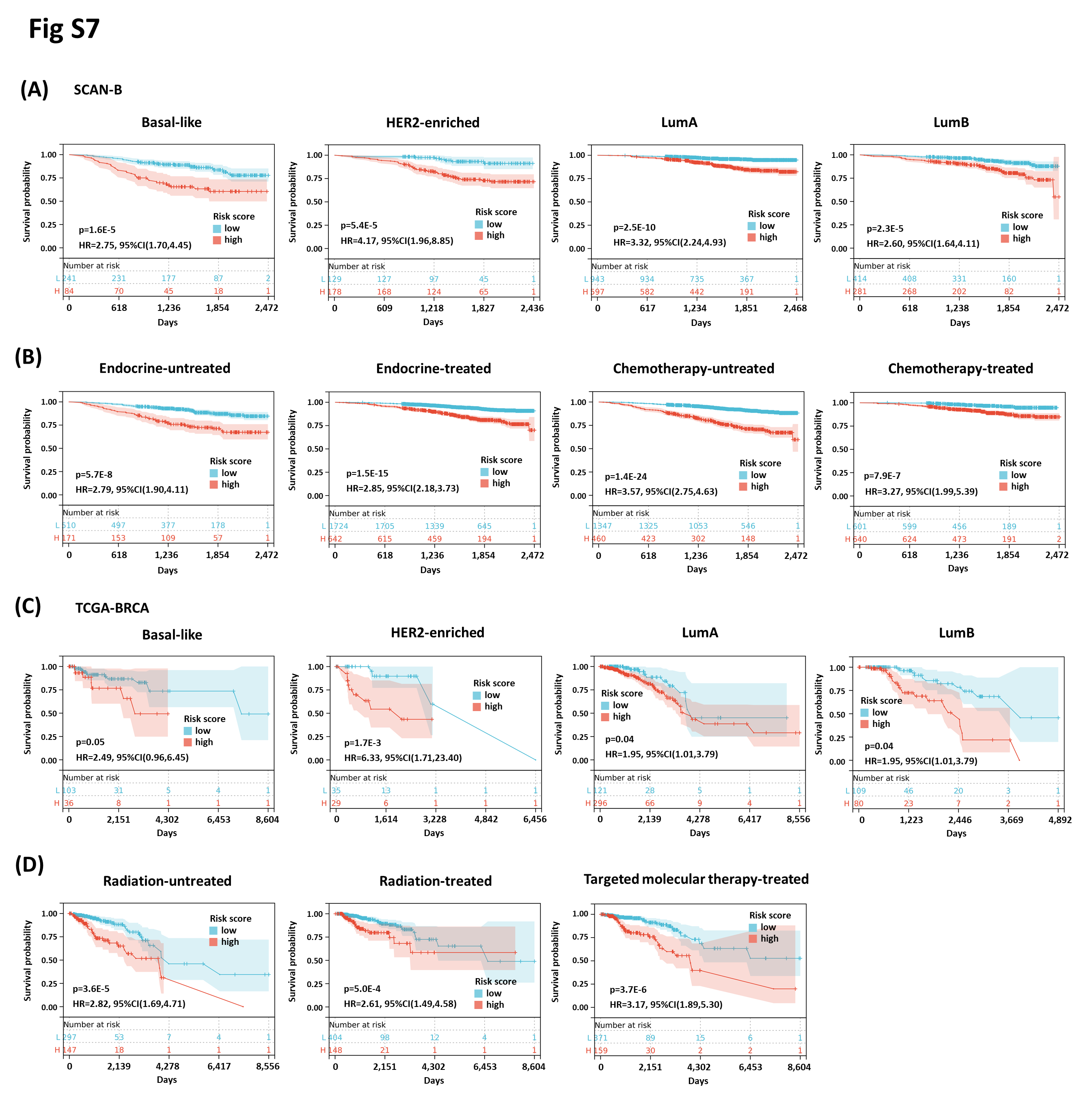


**Figure S7.** Risk score as a prognostic factor in molecular subtypes and different treatment groups. Kaplan‒Meier curves of all the PAM50 subtypes from the SCAN-B (A) and TCGA-BRCA (C) cohort. (B) Kaplan‒Meier curves of endocrine-untreated, endocrine-treated, chemotherapy-untreated and chemotherapy-treated groups in the SCAN-B cohort. (D) Kaplan‒Meier curves of radiation-untreated, radiation-treated and targeted molecular therapy-treated groups in the TCGA-BRCA cohort. P values were calculated via the log-rank test.


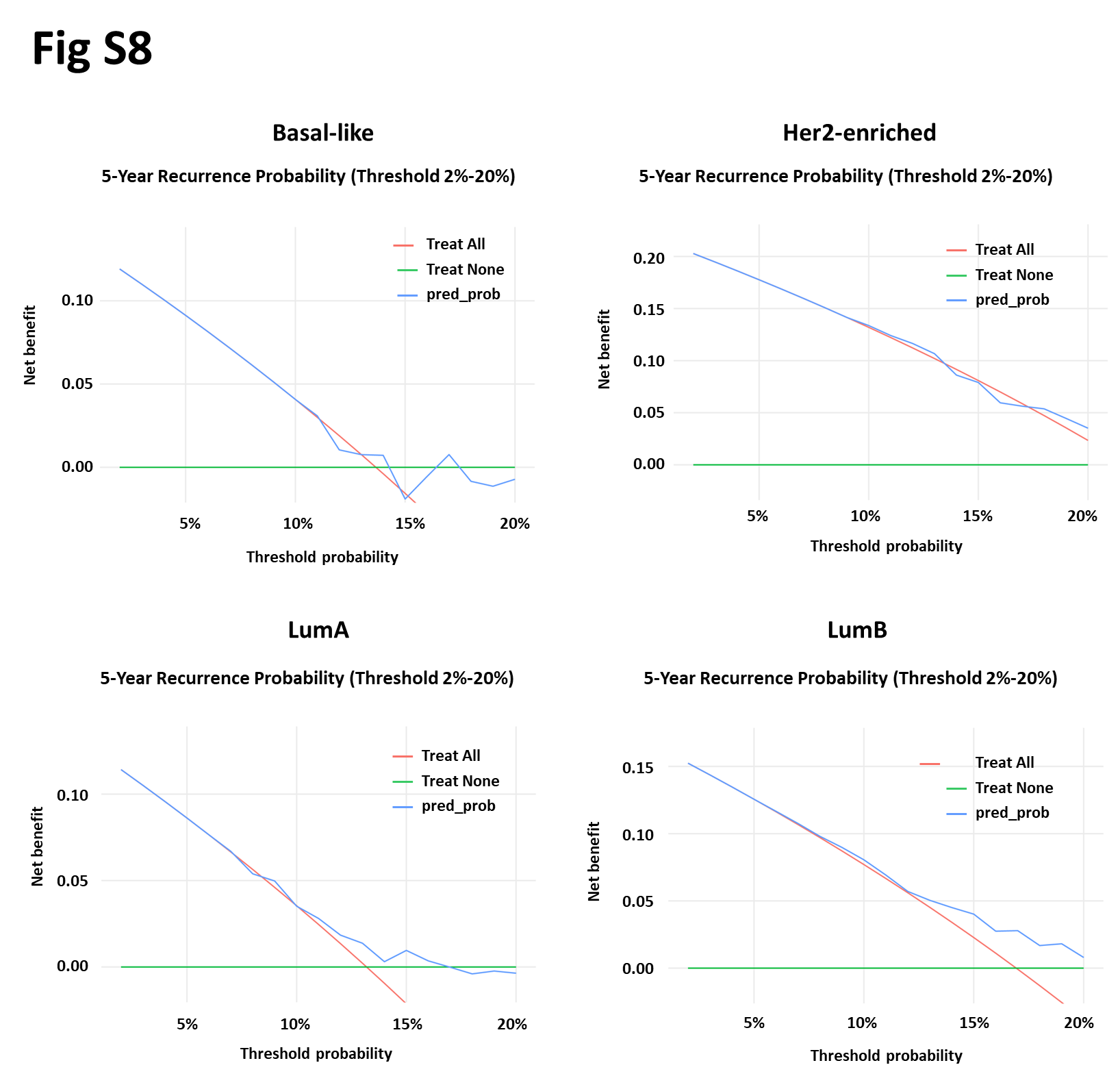


**Figure S8.** Decision curve analysis (DCA) of the prognostic model in molecular subtypes from the TCGA-BRCA cohort.


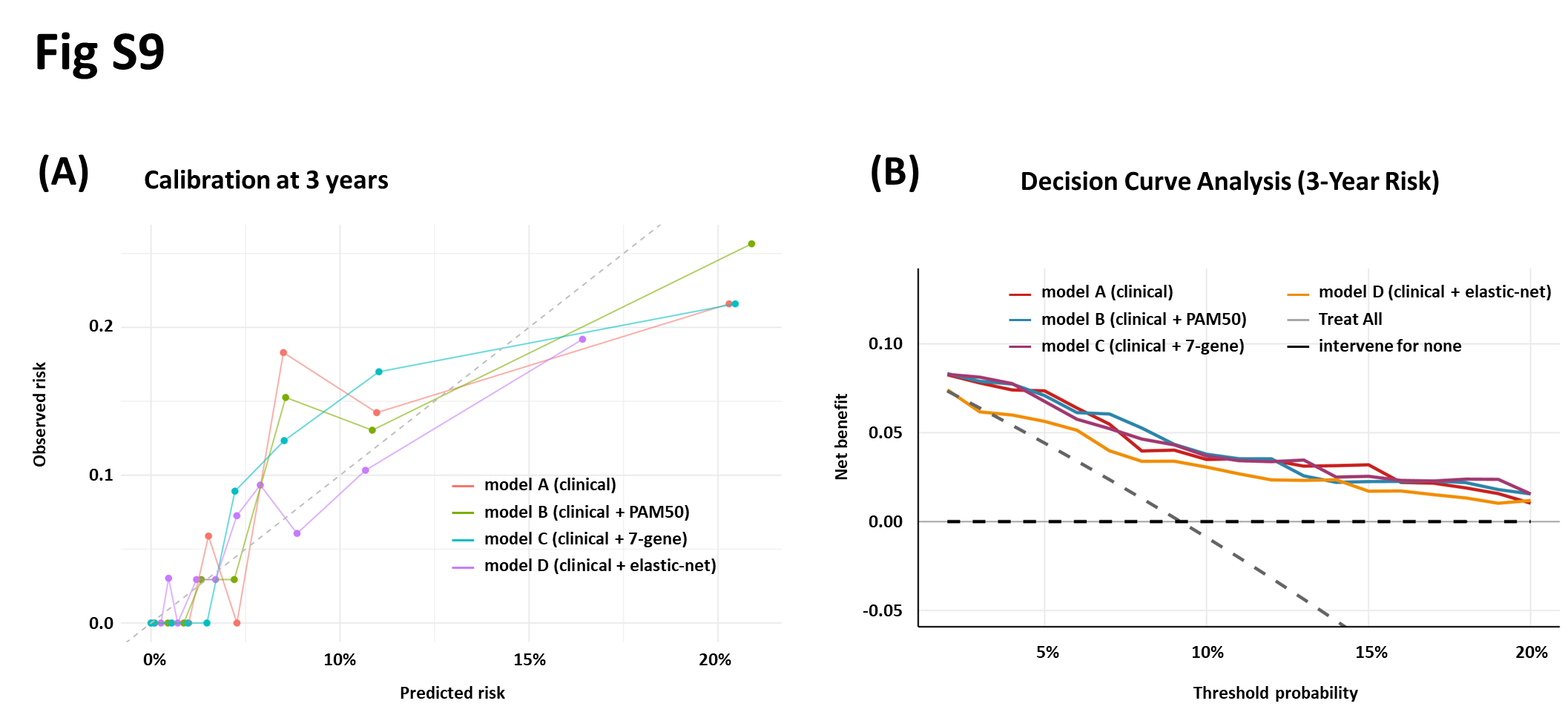


**Figure S9.** Evaluation of four prognostic models. (A) Overlay Plot of Calibration Curves of four prognostic models in the SCAN-B cohort. (B) DCA of four prognostic models.


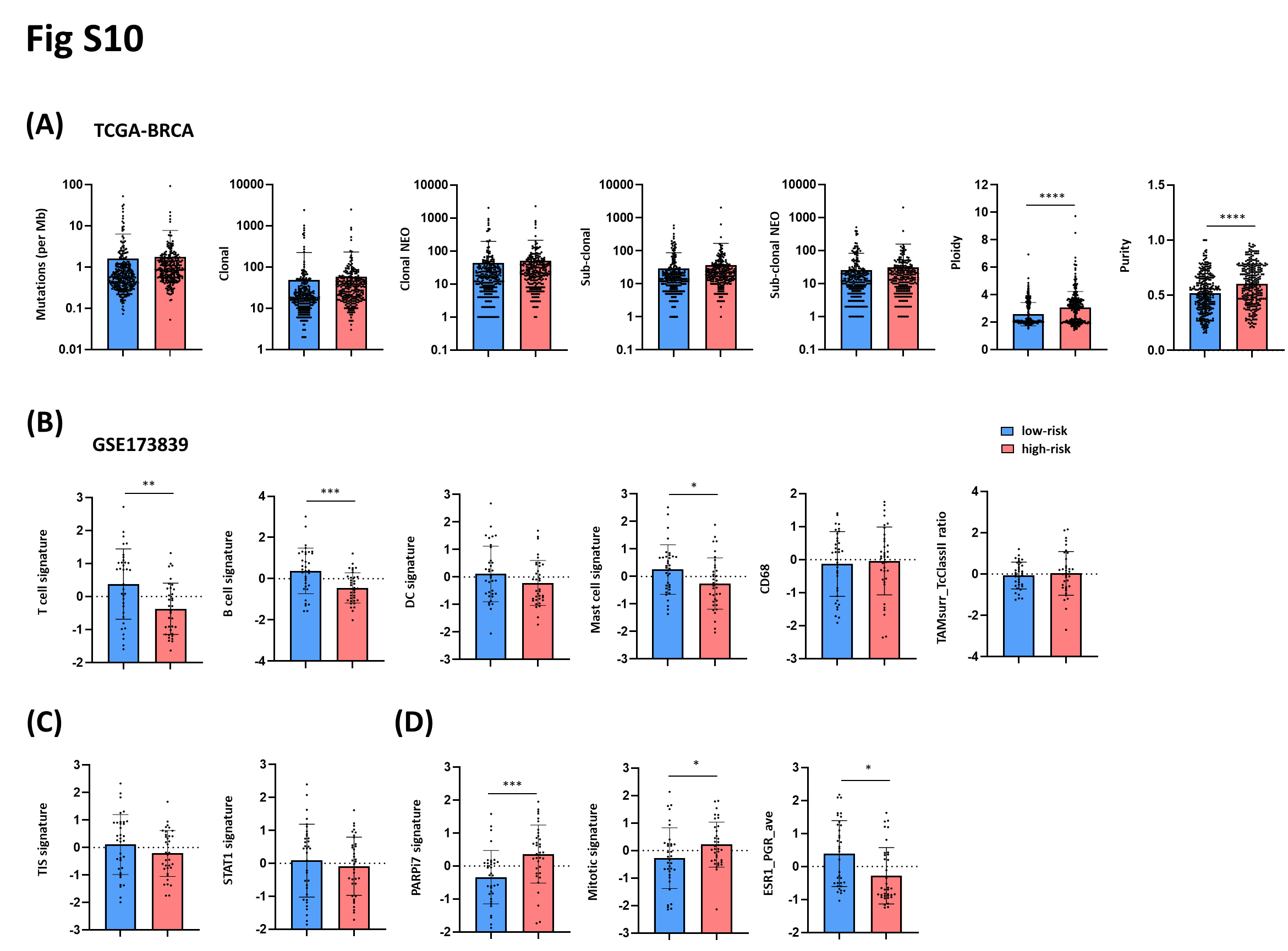


**Figure S10.** Immune landscape in the low-and high-risk groups. (A) Mutation ratios, clonal mutation, clonal neoantigen, sub-clonal mutation, sub-clonal neoantigen, ploidy and purity in the low-risk (n=303) and high-risk (n=268) groups from the TCGA-BRCA cohorts. (B) Signature of T cells, B cells, DCs, mast cells, macrophages, and TAMsurr/TcClassII in the low-risk (n=35) and high-risk (n=36) groups from GSE173839. (C) Signature of TIS and STAT1. (D) PARPi7 signature, mitotic signature, and ESR1_PGR_ave. The data are presented as the means ± SDs. P values were calculated via Student’s unpaired t test. *, P < 0.05; **, P < 0.01; ***, P < 0.001; ****, P < 0.0001.


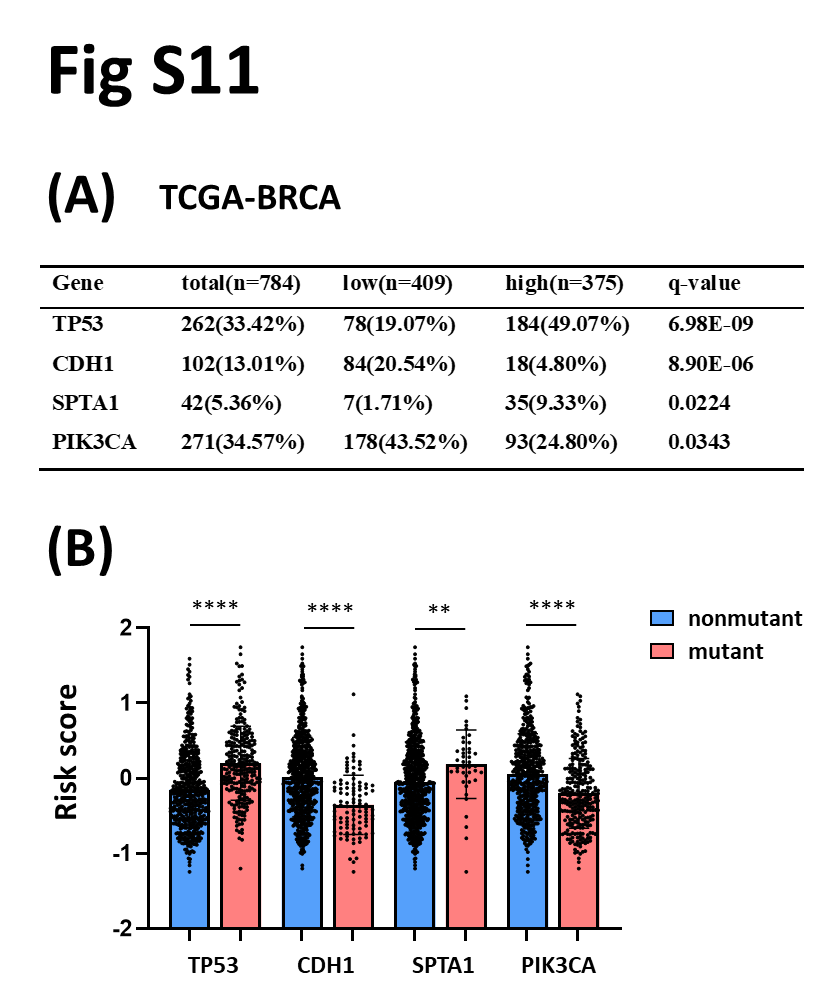


**Figure S11.** Gene mutations in the low- and high-risk groups. (A) List of mutation frequencies of four genes in the TCGA-BRCA cohorts. (B) The levels of risk scores in nonmutant and mutant groups of four genes. The data are presented as the means ± SDs. Q values were obtained by first calculating p values using the chi-square test, followed by adjustment with the Benjamini-Hochberg method in A. P values were calculated via Student’s unpaired t test in B. **, P < 0.01; ****, P < 0.0001.

**
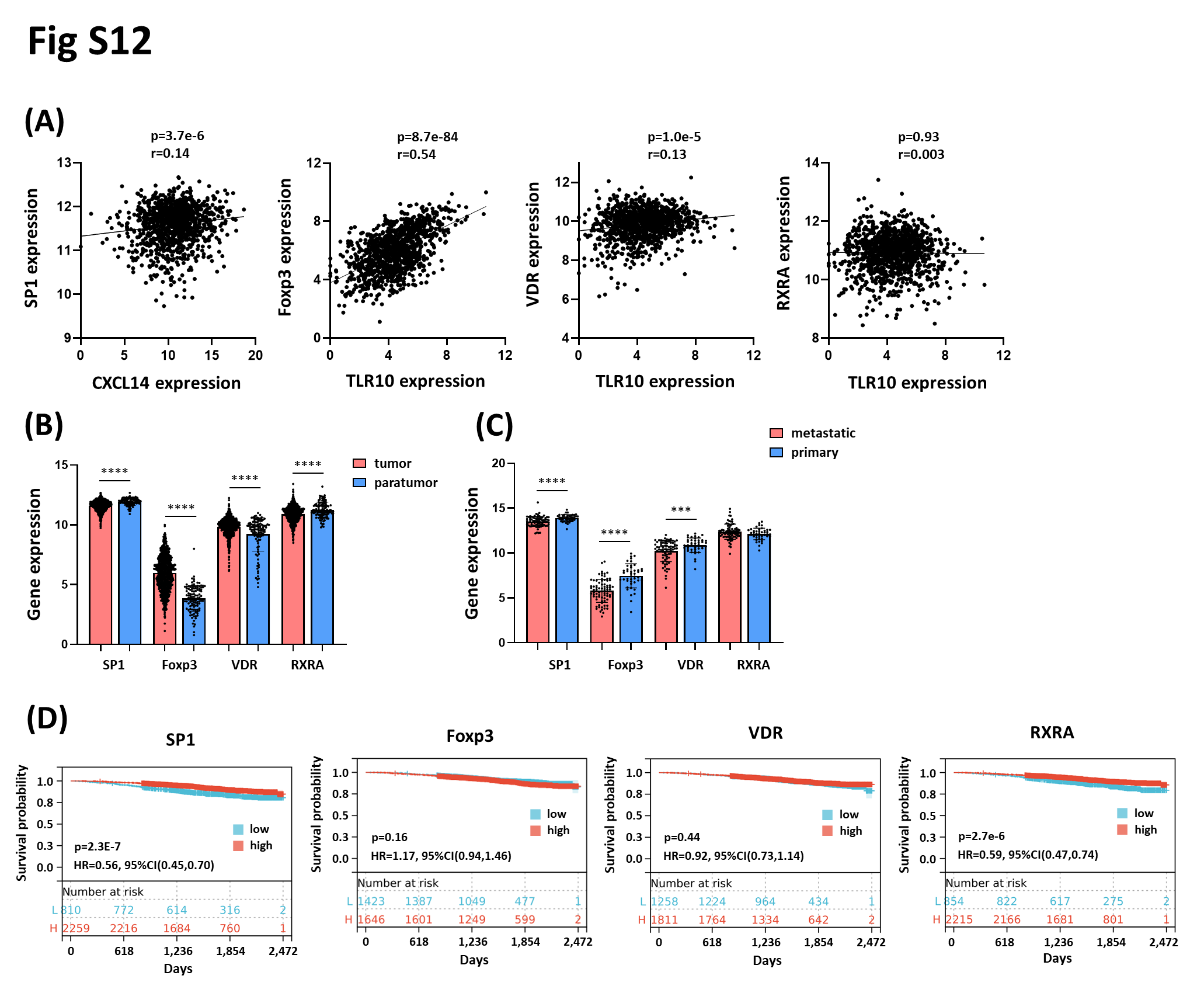
**

**Figure S12.** Network analysis of TFs and prognostic genes. (A) Correlations of TF genes and prognostic genes in the TCGA-BRCA cohort (n=1097). (B) Expression of TF genes in tumor (n=1097) and paratumor (n=114) samples from TCGA-BRCA. (C) Expression of TF genes in metastatic (n=79) and primary (n=44) tumors from the AURORA US Network. (D) Kaplan‒Meier curve of TF genes in SCAN-B. The data are presented as the means ± SDs. P values were calculated via linear regression in A, Student’s unpaired t test in B and C, and the log-rank test in E. ***, P < 0.01; ****, P < 0.0001.
